# Characteristics of linkage disequilibrium in North American Holsteins

**DOI:** 10.1186/1471-2164-11-421

**Published:** 2010-07-08

**Authors:** Jarmila Bohmanova, Mehdi Sargolzaei, Flavio S Schenkel

**Affiliations:** 1 Centre for Genetic Improvement of Livestock, Animal and Poultry Science, Department, University of Guelph, Guelph, Ontario, Canada; 2 L'Alliance Boviteq, Saint-Hyacinthe, Québec, Canada

## Abstract

**Background:**

Effectiveness of genomic selection and fine mapping is determined by the level of linkage disequilibrium (LD) across the genome. Knowledge of the range of genome-wide LD, defined as a non-random association of alleles at different loci, can provide an insight into the optimal density and location of single-nucleotide polymorphisms (SNPs) for genome-wide association studies and can be a keystone for interpretation of results from QTL mapping.

**Results:**

Linkage disequilibrium was measured by |D'| and r^2 ^between 38,590 SNPs (spaced across 29 bovine autosomes and the X chromosome) using genotypes of 887 Holstein bulls. The average level of |D'| and r^2 ^for markers 40-60 kb apart was 0.72 and 0.20, respectively in Holstein cattle. However, a high degree of heterogeneity of LD was observed across the genome. The sample size and minor allele frequency had an effect on |D'| estimates, however, r^2 ^was not noticeably affected by these two factors. Syntenic LD was shown to be useful for verifying the physical location of SNPs. No differences in the extent of LD and decline of LD with distance were found between the intragenic and intergenic regions.

**Conclusions:**

A minimal sample size of 444 and 55 animals is required for an accurate estimation of LD by |D'| and r^2^, respectively. The use of only maternally inherited haplotypes is recommended for analyses of LD in populations consisting of large paternal half-sib families. Large heterogeneity in the pattern and the extent of LD in Holstein cattle was observed on both autosomes and the X chromosome. The extent of LD was higher on the X chromosome compared to the autosomes.

## Background

The discovery of new SNPs and the continuous declining cost of genotyping the bovine genome are making it possible to investigate genome-wide linkage disequilibrium (LD) in detail. Linkage disequilibrium refers to a non-random association in the occurrence of alleles at two loci, which results in a higher frequency of certain haplotypes at the loci than expected by chance.

The knowledge of the extent and the pattern of LD throughout the bovine genome plays an important role in gene mapping and genome-wide association studies and is a fundamental tool for: exploring the degree of diversity among breeds of cattle, inferring the distribution of crossing-over, and identifying regions of genome that have been subject to selective sweep. A high resolution LD map can also provide beneficial information for designing SNP panels for genome-wide selection.

The two most commonly used measures of LD for bi-allelic markers are r^2 ^and |D'|. The range of both measures is between 0 and 1. |D'| < 1 indicates that historical recombination has occurred between the loci and |D'| = 1 indicates absence of recombination between the two loci since the occurrence of one of the polymorphism. Therefore |D'| is rather an indicator of missing haplotypes than a reliable measure of LD [1]. Moreover |D'| tends to be strongly inflated in small samples and in the presence of a rare allele [2]. r^2 ^represents the correlations between the two loci and r^2 ^= 1 when only two haplotypes are present, which is usually a consequence of population bottlenecks or genetic drift [3]. r^2 ^is preferred for association studies, because there is a simple inverse relationship between r^2 ^and the sample size that is required to detect the association between a QTL and the SNP [4].

Diploid cells of *Bos taurus *contain 29 homologous autosomal pairs and one pair of sex chromosomes. All autosomes are acrocentric, however, both gonosomes (X and Y) are submetacentric [5]. The X chromosome (Chr X) is the second largest chromosome of the bovine karyotype [6] and is composed of two distinct regions: the pseudo-autosomal region (PAR), which is homologous to the Y chromosome and the X-specific region. In females, the cross-over can occur at any segment of the Chr X. On the other hand, in males, the cross-over is limited only to the PAR region. There is also variability in recombination rates on autosomes [7], which, apart from other factors, leads to considerable diversity of the LD pattern in different genomic regions. Generally, LD is higher on the Chr X than on the autosomes [8]. The Y chromosome is one of the smallest chromosomes [6], its Y-specific part is gene poor and consists of repetitive sequences, which makes a physical mapping of this region difficult and consequently there is no Y chromosome map available so far for bovine [9].

Previous studies on LD in dairy cattle were based on microsatellites and reported high levels of LD for long distances [10-12]. Recent studies used SNPs for evaluating the extent of LD and revealed that strong LD extends for shorter distances than previously reported. Marques et al. [13] reported (analyzing 505 SNPs on chromosome 14 in Holsteins) moderate levels of LD (r^2^≥0.2) extending up to 100 kb, similar results were presented by McKay et al. (2,670 SNPs) [14]. In the study by Khatkar et al. (9,195 SNPs) [15], r^2^≥0.2 was observed in Australian Holsteins between SNPs less than 60 kb apart. Sargolzaei et al.[16] reported useful LD (r^2^≥0.2) between markers with inter-marker distance ≤ 100 kb in North American Holsteins (5,564 SNPs). There is variation in the published extent of LD because the estimates of LD strongly depends on various factors such as: history and structure of the studied population (evolutionary forces that affected the population), sample size, marker type (microsatellites or SNPs), density and distribution of markers, type of method used for haplotype reconstruction, strictness of SNP filtering (threshold of minor allele frequencies and Hardy-Weinberg equilibrium), use of maternal haplotypes only or both maternal and paternal haplotypes. The objective of this study was to investigate the extent and the pattern of LD on the autosomes and on the chromosome X of North American Holsteins.

## Results and discussion

**Figure 1:**
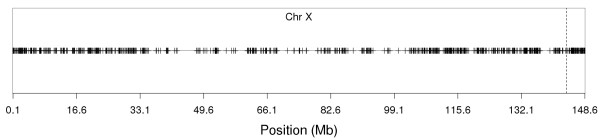
**Distribution of SNPs across Chr X**. The dashed line depicts the boundary between the X-specific and the pseudo-autosomal regions.

Out of 38,590 SNPs used in this study, 37,899 SNPs were spread out across the 29 bovine autosomes (BTA) and 691 SNPs were located on Chr X. The approximate boundary between the X-specific and PAR regions was located at 143.83 Mb, which is in agreement with Sandor et al. [8]. The X-specific and the PAR regions harboured 629 and 62 SNPs, respectively. The distribution of SNP minor allele frequencies (MAF) of these SNPs was nearly uniform on both autosomes and Chr X (Additional file 1, Figure S1). The average MAF of all SNPs before editing was 0.26 and after the 4,446 SNPs with MAF < 0.05 were removed the averaged MAF increased to 0.29. The average MAF was slightly higher than the average MAF of SNPs reported for the SNPs on the Illumina BovineSNP50K BeadChip for Holstein by the Bovine Hapmap Consortium (0.25) [17] and by Matukumalli et al. (0.26) [18]. The SNPs were almost uniformly distributed across the autosomes with an average inter-marker distance of 66 kb (Additional file 2, Figure S2). On the other hand, SNPs were not evenly spaced on Chr X (Additional file 3, Figure S3). There were certain regions of Chr X where adjacent SNPs were separated by more than 1 Mb (Figure 1). The average distance between the adjacent SNPs on Chr X was 215 kb.

### Analysis of decay of linkage disequilibrium

**Figure 2:**
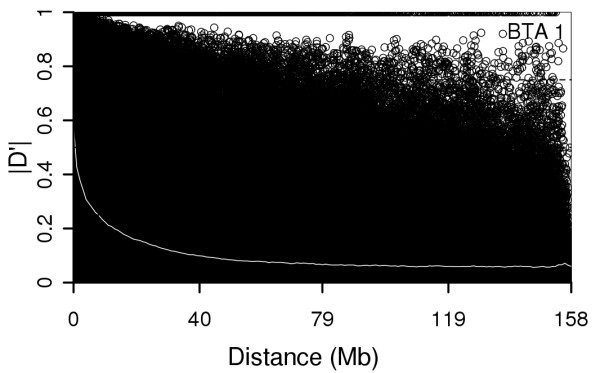
**Decay of |D'| as function of physical distance on BTA 1**. The white line represents the median decline of |D'|.

In order to examine the decay of LD with physical distance, SNP pairs on the autosomes were sorted into bins based on their inter-marker distance and average values of r^2 ^and |D'| were calculated for each bin (Table 1 and 2). Moderate levels of r^2 ^(r^2 ^between 0.2 and 0.24) were observed at distances shorter than 60 kb. When moving from 60 to 500 kb, average r^2 ^declined from 0.16 to 0.11. High variability of r^2 ^was observed in bins with a marker distance < 1 Mb. Markers with r^2^≥0.3 were on average spaced by 60 kb. But not all markers with the 60 kb inter-marker distance had r^2^≥0.3. In the 0 to 40 kb and 40 kb to 60 kb bins, only 30 and 23% of markers had r^2 ^≥0.3, respectively. Figure 2 shows a scatter plot of |D'| values against distance. In contrast to decay of r^2^, where LD values clearly declined with distance, the changes of |D'| followed a much less steeper declining pattern. Average |D'| of markers 40-60 kb apart was 0.72 and 19% of SNP pairs with 0.5-1 Mb inter-marker distance had |D'| > 0.8 (Table 2). |D'| = 1 was observed at distances exceeding 100 MB.

**Table 1:** Pairwise linkage disequilibrium (r^2^) for syntetic SNPs at various distanced pooled over all autosomes

Distance	N	Mean	SD	Median	95% CI of the Mean		% (r^2 ^> 0.3)*	% (r^2 ^> 0.15)^#^
0-40 kb	16,237	0.24	0.26	0.24	0.24	0.25	30	47
40-60 kb	12,683	0.20	0.23	0.19	0.19	0.20	23	40
60-100 kb	25,527	0.16	0.20	0.16	0.15	0.16	17	33
100-200 kb	62,422	0.11	0.15	0.11	0.11	0.12	10	24
200-500 kb	183,089	0.08	0.11	0.08	0.08	0.08	5	17
0.5-1 Mb	299,167	0.07	0.09	0.07	0.07	0.07	3	13
1-2 Mb	583,426	0.06	0.08	0.06	0.06	0.06	2	10
2-5 Mb	1,671,022	0.04	0.06	0.04	0.04	0.04	1	5
5-10 Mb	2,584,922	0.03	0.04	0.03	0.03	0.03	0	2
10-20 Mb	4,615,064	0.02	0.02	0.02	0.02	0.02	0	0
20-50 Mb	9,971,401	0.01	0.01	0.01	0.01	0.01	0	0
50-100 Mb	6,658,292	0.00	0.01	0.24	0.24	0.25	0	0
>100 Mb	988,416	0.00	0.00	0.19	0.19	0.20	0	0

*Percentage of pairs of SNPs with r^2^> 0.3, ^#^Percentage of pairs of SNPs with r^2^> 0.15

**Table 2:** Pairwise linkage disequilibrium (|D'|) of syntenic SNPs at various distanced pooled over all autosomes

Distance	N	Mean	SD	Median	95% CI of the Mean		% Strong LD*
0-40 kb	16,237	0.76	0.31	0.94	0.76	0.77	62
40-60 kb	12,683	0.72	0.32	0.88	0.71	0.72	53
60-100 kb	25,527	0.67	0.33	0.78	0.66	0.67	43
100-200 kb	62,422	0.62	0.33	0.67	0.61	0.62	33
200-500 kb	183,089	0.56	0.32	0.58	0.56	0.56	24
0.5-1 Mb	299,167	0.52	0.31	0.52	0.52	0.52	19
1-2 Mb	583,426	0.48	0.30	0.46	0.48	0.48	14
2-5 Mb	1,671,022	0.41	0.28	0.38	0.41	0.41	8
5-10 Mb	2,584,922	0.33	0.24	0.28	0.33	0.33	3
10-20 Mb	4,615,064	0.24	0.19	0.19	0.24	0.24	1
20-50 Mb	9,971,401	0.15	0.14	0.11	0.14	0.14	0
50-100 Mb	6,658,292	0.10	0.10	0.07	0.10	0.10	0
> 100 Mb	988,416	0.09	0.09	0.06	0.09	0.09	0

*Percentage of pairs of SNPs with |D'| > 0.8

On Chr X, LD was calculated separately for SNPs located on the X-specific and the PAR regions of Chr X (Table 3). Higher levels of LD were found on the X-specific region of Chr X than on autosomes. Very low levels of LD, even at very short distances (<40 kb), were reported on the PAR region. This was expected because in males, the cross-over is limited to the PAR region and consequently the rate of recombination was higher in this segment. On the other hand, the X-specific region is recombined only in gametes produced by females and consequently only two thirds of the X chromosomes recombine in each generation [19]. Overall, LD on Chr X was greater compared to LD on autosomes. It can be explained by not only a lower recombination rate but also by a lower mutation rate and a faster genetic drift due to a smaller effective population size [19].

**Table 3:** Pairwise linkage disequilibrium (r^2^) for syntetic SNPs at various distanced pooled over X-specific and pseudo-autosomal (PAR) regions of the X chromosome

Distance	X-specific			Pseudo-autosomal		
		
	N	Mean	SD	N	Mean	SD
0-40 kb	126	0.28	0.28	24	0.15	0.18
40-60 kb	106	0.23	0.26	18	0.08	0.10
60-100 kb	197	0.18	0.24	32	0.10	0.14
100-200 kb	461	0.11	0.17	89	0.05	0.07
200-500 kb	1,184	0.08	0.13	262	0.06	0.07
0.5-1 Mb	1,755	0.06	0.09	361	0.04	0.05
1-2 Mb	3,144	0.05	0.07	584	0.03	0.04
2-5 Mb	8,903	0.04	0.06	-	-	-
5-10 Mb	13,678	0.04	0.05	-	-	-
10-20 Mb	23,588	0.03	0.04	-	-	-
20-50 Mb	46,808	0.02	0.03	-	-	-
50-100 Mb	59,598	0.01	0.01	-	-	-
> 100 Mb	36,703	0.01	0.01	-	-	-

Table 4 shows mean r^2 ^and |D'| of the 30 individual *Bos taurus *chromosomes and mean r^2 ^and |D'| of adjacent SNPs pooled over all 29 autosomes and Chr X. The average recombination distance in bovine genome is 1.25 cM per Mb but the recombination distances decrease with the length of the chromosome and therefore the rate of recombination increases with the length of the chromosome [7]. This suggests that in general, LD of longer chromosomes will extend for shorter distances and consequently such chromosomes will have lower overall LD than shorter chromosomes. In this study, certain chromosomes had higher LD than others but there was no relationship between the length of the chromosome or the average spacing of SNPs and the extent of the LD. The average r^2 ^ranged from 0.17 (BTA27, BTA28 and BTA 29) through 0.25 (BTA 7 and BTA14) and |D'| ranged from 0.59 (Chr X) through 0.73 (BTA1, BTA 3 and BTA 8). The average MAF per chromosome ranged from 0.28 to 0.30.

**Table 4:** Adjacent linkage disequilibrium (r^2 ^and |D'|) on 29 autosomes (BTA)

Chromosome	N	Length (Mb)	Spacing (kb)	Mean r^2^	SD (r^2^)	Median r^2^	Mean |D'|	SD |D'|	Median |D'|	MAF
1	2,443	158	64	0.22	0.25	0.11	0.73	0.32	0.90	0.28
2	1,970	136	69	0.22	0.25	0.11	0.70	0.33	0.85	0.29
3	1,859	121	65	0.22	0.25	0.12	0.73	0.33	0.90	0.29
4	1,804	120	66	0.20	0.23	0.10	0.70	0.34	0.86	0.29
5	1,603	121	75	0.20	0.24	0.10	0.71	0.33	0.87	0.29
6	1,848	119	64	0.21	0.25	0.11	0.70	0.33	0.85	0.29
7	1,615	112	69	0.23	0.25	0.13	0.71	0.32	0.86	0.29
8	1,721	113	65	0.21	0.25	0.11	0.73	0.32	0.89	0.28
9	1,487	105	70	0.20	0.23	0.10	0.70	0.33	0.85	0.28
10	1,559	104	66	0.21	0.24	0.12	0.71	0.32	0.84	0.29
11	1,654	107	64	0.20	0.23	0.10	0.70	0.33	0.85	0.29
12	1,260	91	72	0.20	0.24	0.10	0.70	0.33	0.84	0.28
13	1,319	84	63	0.21	0.24	0.12	0.72	0.33	0.90	0.30
14	1,326	84	62	0.23	0.26	0.13	0.72	0.32	0.88	0.30
15	1,267	85	66	0.19	0.23	0.09	0.70	0.33	0.87	0.29
16	1,166	81	69	0.22	0.25	0.12	0.72	0.32	0.88	0.29
17	1,194	74	62	0.19	0.23	0.10	0.72	0.32	0.87	0.29
18	1,013	65	64	0.18	0.22	0.09	0.66	0.34	0.78	0.30
19	1,039	64	61	0.19	0.23	0.10	0.70	0.33	0.85	0.29
20	1,126	71	63	0.21	0.23	0.12	0.72	0.31	0.85	0.29
21	1,031	71	69	0.20	0.24	0.10	0.68	0.33	0.81	0.30
22	965	61	63	0.19	0.25	0.09	0.69	0.33	0.82	0.28
23	818	52	63	0.18	0.22	0.09	0.64	0.35	0.74	0.30
24	922	62	67	0.20	0.24	0.10	0.68	0.33	0.82	0.29
25	770	42	55	0.20	0.23	0.11	0.68	0.33	0.79	0.30
26	804	51	63	0.18	0.24	0.08	0.67	0.34	0.82	0.28
27	745	45	60	0.17	0.22	0.07	0.66	0.34	0.77	0.29
28	731	46	63	0.17	0.20	0.09	0.65	0.34	0.73	0.30
29	811	51	63	0.17	0.22	0.09	0.68	0.33	0.79	0.29
X	690	148	215	0.18	0.23	0.08	0.59	0.33	0.63	0.29
All	38,560	2656	68	0.20	0.24	0.11	0.70	0.33	0.85	0.29

Linkage disequilibrium of intragenic (within genes) and intergenic pairs of SNP was investigated. Kim et al. [20] reported a higher LD and a higher variation in intragenic regions than in intergenic regions. In this study, there were no differences in the extent of LD and the decline of LD with the distance between intragenic and intergenic regions (Table 5).

**Table 5:** Linkage disequilibrium (r^2^) of intragenic and intergenic pairs of SNP

	Intergenic			Intragenic		
		
Distance (kb)	N	Mean	SD	N	Mean	SD
< 40	13,302	0.21	0.06	2,849	0.20	0.06
40-60	7,037	0.20	0.06	1,055	0.19	0.06
60-80	4,273	0.20	0.06	516	0.21	0.06
80-100	2,716	0.20	0.06	259	0.20	0.06

**Figure 3:**
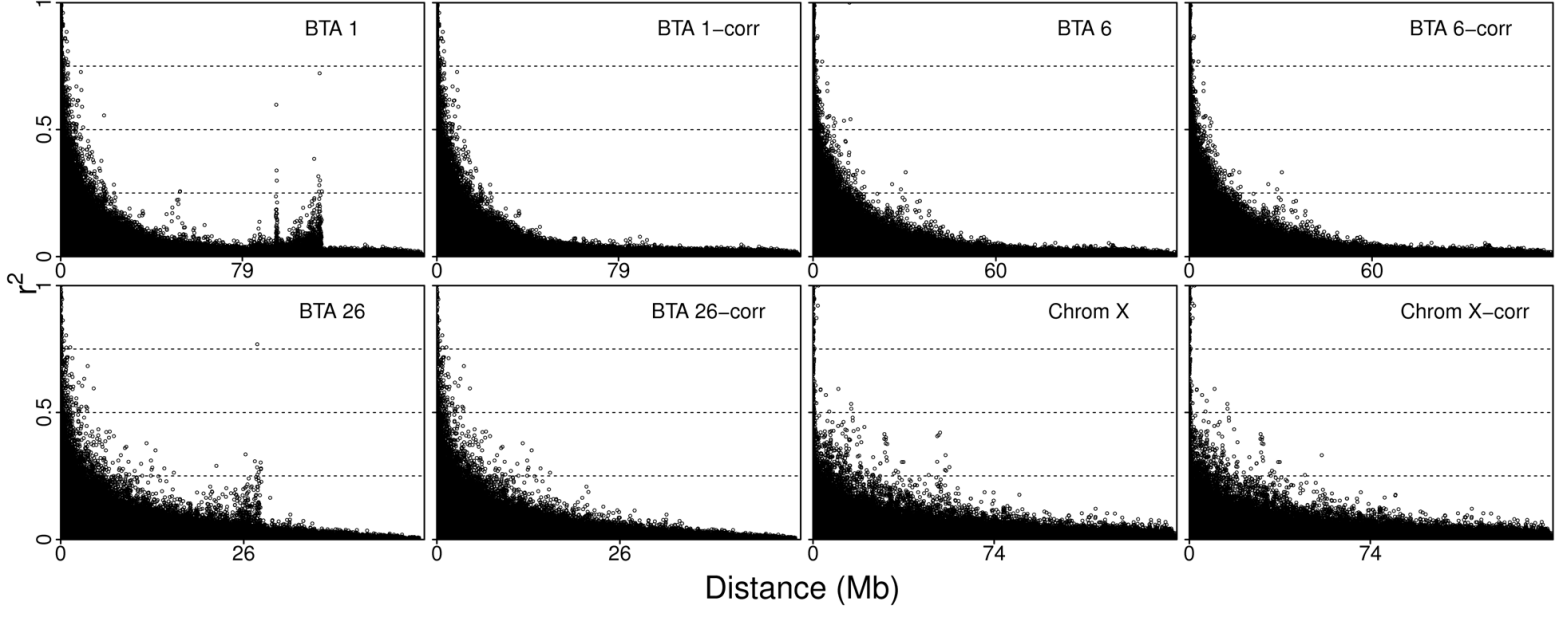
**Decay of r^2 ^as function of physical distance**. The plots represent pairwaise LD on chromosomes 1, 6, 26 and X before and after (corr) positions of the misplaced SNPs were corrected.

When the decay of r^2 ^with a distance was plotted separately for each chromosome, several SNP pairs on BTA1, BTA6, BTA 26 showed higher r^2 ^than expected (Figure 3). This was a possible indication of misplaced SNPs on those chromosomes. In order to identify those likely misplaced SNPs, a simple algorithm based on investigation of the decay of LD with distance was developed. The distance between each of the 38,590 SNPs and a SNP with which it had the largest r^2 ^was recorded. If this distance was larger than 10 MB, the particular SNP was flagged as a possibly misplaced SNP and it was further investigated. This algorithm detected 223 misplaced SNPs. The majority of the misplaced SNPs were on BTA 1 (27), BTA 6 (67), BTA 26 (18) and Chr X (55). The correct location of these SNPs was approximated by assuming that the SNP was located between two markers with the highest pairwise r^2 ^with the SNP. Additional file 4, Figure S4 shows the decay of LD with a distance of using their original location and the corrected position of the misplaced SNPs. The smoother pattern of the declining trend of r^2 ^with a distance in plots where the position of the SNP was corrected suggests that our simplified approximation of the SNP location has a reasonable accuracy. Also the pattern of the decay of LD on BTA1, BTA6, BTA 26 acquired the expected shape after the position of misplaced SNPs was corrected (Figure 3). Our simple algorithm identified 0.57% of SNPs as misplaced. It is very likely that there could be other SNP with incorrect location, especially on BTA 14 and Chr X, where the pattern of LD was slightly erratic in certain regions even after the corrections. This is because the algorithm was not able to identify SNPs that were misplaced by only a few MB, due to the high heterogeneity of LD at short distances.

### Paternal versus maternal haplotypes

Using only maternally inherited haplotypes is a common practice in studies investigating LD with the sample population consisting of large sire half-sib families. The reasoning is that pedigree structure leads to overrepresentation of the paternal haplotypes in the sample, because sires have multiple progeny in the data set, which inflates frequencies of certain haplotypes and consequently leads to overestimation of LD. In order to test this hypothesis and to quantify the size of the bias of LD, |D'| and r^2 ^were calculated using maternal, paternal and both haplotypes. First of all, differences in haplotype frequencies between maternal and paternal haplotypes were tested by a χ^2 ^test. The frequencies of maternal haplotypes were assumed to be the true expected values and they were contrasted to the frequencies of paternal haplotypes. Using the χ^2 ^test, 82% of SNP pairs were identified to have significantly different (P < 0.01) haplotype frequency in maternal than in paternal haplotype. This could be due to the previously mentioned pedigree structure of our sample or it could be a result of different selection pressure in the sire of sires pathway compared to the dam of sires pathway.

**Figure 4:**
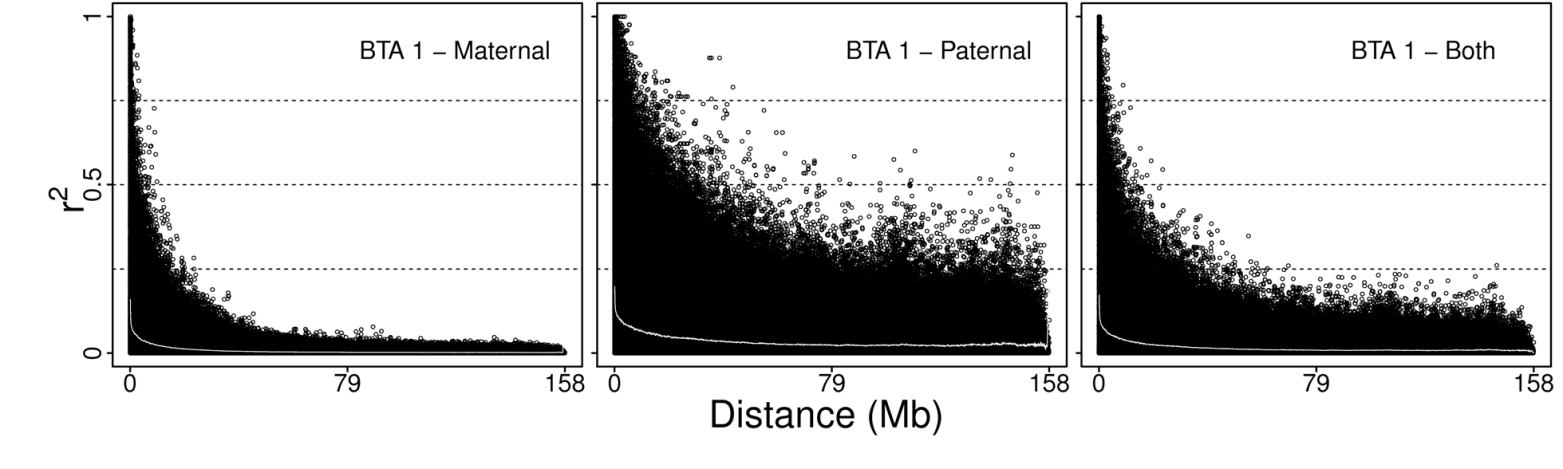
**Comparison of decay of linkage disequilibrium with distance (r^2^) on BTA1 using only maternal, paternal or both haplotypes**. The white line represents the average decline of linkage disequilibrium.

Figure 4 compares the decay of linkage disequilibrium with distance on BTA 1 when r^2 ^was computed from maternal, paternal or both haplotypes. It is quite apparent from these three diagrams that LD decayed slower in paternal haplotypes. Moreover, 49 SNP pairs with 50-100 MB inter-marker distance had noticeably inflated values of paternal r^2 ^(r^2 ^> 0.25). Considering both haplotypes, the negative effect of paternal haplotype was reduced, but a certain bias was still present. Therefore to avoid a possible bias in the estimates of LD due to the pedigree structure, use of only maternal haplotypes is recommended.

### Non-syntenic linkage disequilibrium

Linkage disequilibrium was calculated for all possible combination of pairs of SNPs located on different chromosomes. None of the non-syntenic SNP pairs had r^2 ^> 0.25, which could have occured due to selection or if one of the SNPs was located on a wrong chromosome. 1,444,163 non-syntenic SNP pairs had |D'|≥0.8 but because 94% of these SNPs had MAF < 0.10, it suggests that low MAF largely affected the estimates of |D'|. Low MAF increases chances of a missing haplotype and if at least one haplotype is missing |D'| = 1. On the other hand, SNPs with rare alleles did not have inflated values of r^2^. This is consistent with previous studies [15,16]

### Effect of sample size and MAF on distribution of r^2 ^and |D'|

**Figure 5:**
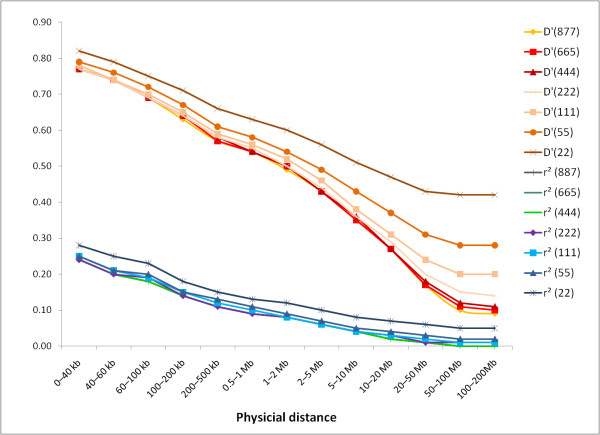
**Distribution of average pairwise r^2 ^and |D'| by sample size and distance**. The number in parenthesis in the legend indicates the sample size of each subset.

As illustrated in Figure 5, the sample size did not have a large effect on r^2 ^values, however, an upward bias was observed in |D'|. This bias increased as the size of the subsample decreased and as the distance between SNPs increased. With the 55 sample size, |D'| was higher by 7% in the 0.5-1 MB distance than with the full data set. Samples with more than 444 bulls had a minimal bias of |D'|. Estimates of r^2 ^were not influenced by the sample size when at least 55 animals were used for the calculation. Samples with only 22 animals resulted in an overestimation of r^2^. These results indicate that a sample size of at least 444 and 55 animals is necessary for an accurate estimation of |D'| and r^2^, respectively. This is in agreement with Khatkar et al. [15] who suggested a minimal sample size of 400 and 75 for estimation of |D'| and r^2^, respectively.

**Figure 6:**
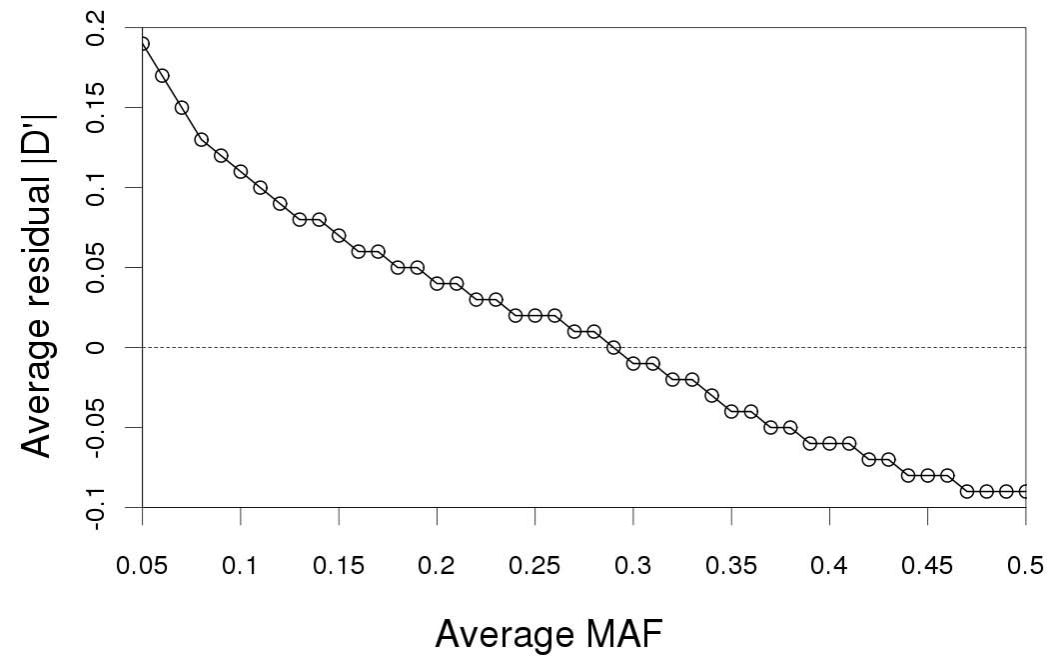
**Average residual |D'| as a function of average MAF of a SNP pair**.

Figure 6 shows residual |D'| as a function of average MAF. Residual |D'| was the highest at a low MAF, suggesting that |D'| is overestimated at low MAF. Bias of |D'| was small (0-0.05) at intermediate MAF (0.2-0.4). Residual |D'| was negative at a high MAF, indicating that |D'| is underestimated at high MAF. This is not a surprising finding, because the denominator in the formula of |D'| is equal to a minimal product of SNP allele frequencies. In SNP pairs with low allele frequency, D will be in the formula divided by a small number, resulting in a large |D'|. The opposite is true for pairs with a high allele frequency. Residual r^2 ^was on the other hand independent of the average MAF.

### Patterns of linkage disequilibrium

**Figure 7:**
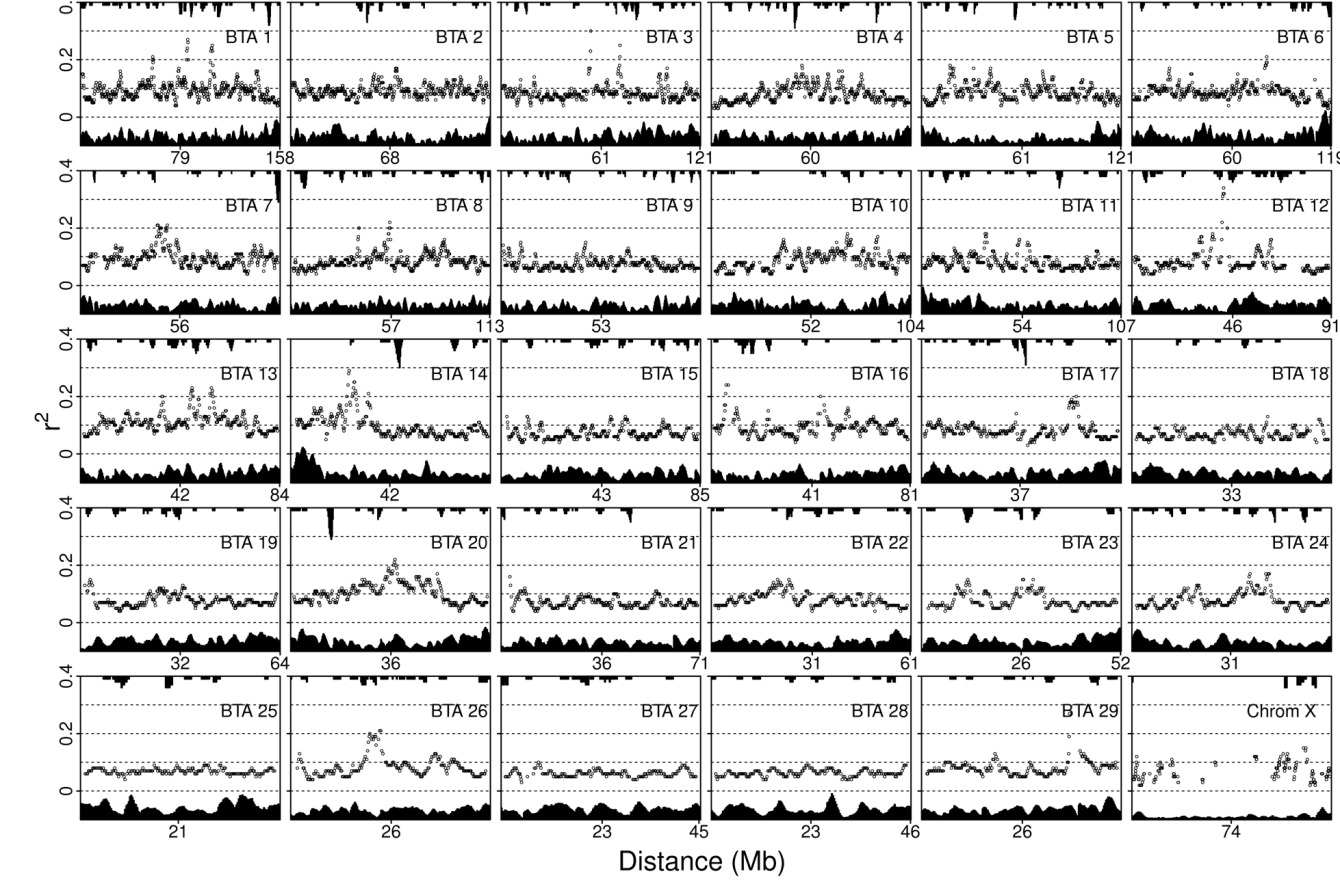
**Average r^2 ^per sliding window (scatter plot), number of SNPs per window (bottom bar plot) and frequency of SNPs with MAF < 0.1 per window (top bar plot)**.

Non-monotonic erratic trend of average r^2 ^was observed in each of the studied 30 chromosomes using the sliding window approach (Figure 7). The erratically high r^2 ^in some sliding windows of autosomes were not caused by a low number of SNPs per window or due to high number of SNPs in the window with MAF < 0.1 probably created by a variation in recombination rates within chromosomes. On the other hand, there were several windows on Chr X that did not even have the required 20 pairs of SNPs.

Chromosomes contain local hot spots and cold spots that undergo quite different rates of meiotic recombination and these spikes in r^2 ^could indicate cold spots. Bovine autosomes are acrocentric and both X and Y chromosomes are subemetacentric [21]. Centromeres are special structures of eukaryotic chromosomes that hold sister chromatids together and ensure proper chromosome segregation during cell division. Centromere suppresses meiotic recombination within itself and also in the proximal chromosomal region. On the other hand, regions next to the telomeres (terminal ends of chromosomes) increase recombination rates [22]. Values of r^2 ^were not noticeably higher at the very beginning or end of any autosomes compared to the middle of the chromosome, as was expected since centromeres are located in *Bos taurus *on telomeres. Therefore this finding suggests that the effect of the centromere and telomeres on recombination rate cancelled one another out. Chr X has centromere in the middle of the chromosome but because there were not enough informative windows in this region, it was not possible to investigate the effect of centromers on the extent of LD of this chromosome. The telomeric parts of the chromosome did not have a noticeable higher r^2 ^than the rest of the chromosome.

## Conclusions

This study shows that |D'| is strongly inflated in small sample sizes and is strongly dependent on the allele frequency. Compared to |D'|, r^2 ^is a more robust measure of LD because it is less sensitive to allele frequency and a small sample size. Because of the widespread use of a few elite sires in dairy cattle, paternally inherited haplotypes are recommended to be discarded, because if they are included in the analyses it could lead to overestimation of the extent of LD. High degree of heterogeneity of LD was observed on both autosomes and Chr X. Higher levels of LD were found on the X-specific region than on the autosomes, the X-specific region also had much higher levels of LD than the PAR region. No differences in the extent of LD and the decline of LD with distance were found between intragenic and intergenic regions. A simple algorithm based on syntenic LD was developed to identify misplaced SNPs and to approximate their physical locations. In total, 223 inconsistencies in physical map of SNPs (built using the UMD 3.1. assembly) were identified but it is very likely that the number of inconsistencies is higher. Because of the high heterogeneity of LD at short distances, our algorithm was able to detect only SNPs that were misplaced by several MB. However, this algorithm can be a useful part in fine mapping, imputation of unknown genotypes, identification of haplotype blocks and selection signatures where correct physical location of SNPs is required. In all these studies, mistakes in the order or position of SNPs can lead to biased results.

## Methods

### Data

The data consisted of 887 Holstein bulls (with both parents born in North America) born from 1990 through 2004 that were genotyped with the Illumina BovineSNP50K BeadChip. All animals had at least 5 progeny or at least 4 brothers in the dataset. The bulls were progeny of 72 sires and 661 dams. The size of the 72 sire families ranged from 1 to 46 bulls per sire and had on average 12 bulls.

The physical map of SNPs was built using the UMD 3.1. bovine genome sequence assembly [23]. SNPs with MAF < 0.05 (4,446), SNPs with more than 10% missing genotypes and SNPs with unknown genomic location in bovine genome assembly were excluded from the analysis. Considering relationship between animals, 0.01% of inconsistencies of SNPs from Mendelian Law were detected and corrected. This editing resulted in 38,590 useful SNPs which were used for further analysis.

### Haplotype phase reconstruction

Haplotypes phases were inferred from pedigree by a method by Sargolzaei et al. [16], which is a family rule based algorithm. Only maternally inherited haplotypes were used for further detailed analyses in order to minimize the effect of over-representation of paternally inherited haplotypes within pedigrees of the bulls.

### Measures of linkage disequilibrium

Linkage disequilibrium between two SNPs was measured using r^2 ^and the absolute value of D'. r^2 ^between SNP pairs with alleles *A *and *a *at the first locus and *B *and *b *at the second locus was defined as , where

and

where π_A_, π_a_, π_B_, π_b _denote the allele frequencies and π_AB_, π_Ab_, π_aB_, π_ab _are the haplotypes frequencies. If the two loci are independent, then the expected frequency of haplotypes AB is π_A _π_B_. If π_AB _is either higher or lower than expected then it indicates that these particular alleles tend to be segregating jointly and are in LD.

### Linkage disequilibrium decay and sliding window analyses

SNP pairs were sorted into bins based on their inter-marker distance and average values of r^2 ^and |D'| were calculated for each bin. 95% confidence intervals of mean of r^2 ^and |D'| were constructed for each bin by a bootstrap analyses [24]. 1,000 samples of the same size as the original sample were randomly sampled with replacement for each bin. The mean value of r^2 ^and |D'| was calculated for each bootstrap dataset and the distribution of all means was used to estimate the confidence intervals.

Sliding windows of 2 Mb (overlapping by 1.9 Mb) were considered for exploring patterns of variation in LD. Only windows consisting of at least 20 SNP pairs were considered. Within each window, average values of r^2 ^and |D'| were calculated for SNPs separated by between 200 to 600 kb.

### Intragenic and intergenic regions

SNP pairs were classified into intragenic and intergenic groups using genomic positions of 21,364 *Bos taurus *genes (Btau 4.0 build) that were obtained from Biomart Martviewer http://www.biomart.org/biomart/martview/. SNP pairs that were located between starting and termination sites of a gene were assigned to the intragenetic group and remaining SNPs were assigned to the intergenetic SNP. Linkage disequilibrium was calculated for each group separately to investigate whether LD within genes is higher or lower than between genes.

### Effect of samples size and MAF on LD

Dependence of |D'| and r^2^on the sample size was demonstrated by taking bootstrap subsamples [2] (with replacement) of size 22, 55, 111, 222, 444, 665 and 877 (1/40, 1/16, 1/8, 1/4, 1/2, 3/4 and 1) from the original dataset. Two hundred replicates were generated for each sample size. The average |D'| and r^2 ^at specific ranges of distances were calculated for each sample size.

The effect of MAF on |D'| and r^2 ^was investigated as suggested by Du et al. [25]. LD is a function of distance, therefore in order to evaluate the effect of MAF on the measures of LD independently of distance, r^2 ^and |D'| between all pairs of SNPs were adjusted for inter-marker distance. The pairs were grouped into bins and the average LD was calculated for each bin, as previously described. Residual LD was calculated for each pair by subtracting average LD of the bin the pair belonged to from the LD of the pair. Further, SNPs were divided into groups based on the average MAF (the average of MAF of the two SNPs). For each MAF group, average residual r^2 ^and |D'| were calculated.

### Boundaries between the X-specific and PAR regions of the chromosome X

The dataset used in this study contained genotypes of bulls only. In bulls, SNPs located in the X-specific region do not have a counterpart on the chromosome Y and consequently these SNPs can have only two genotypes. The PAR region is a terminal segment of both chromosomes X and Y. Therefore SNPs positioned in the PAR region can have up to three different genotypes. The boundary between these two regions was approximated by locating the first SNP in the terminal segment of Chr X, which followed a set of SNPs that had in the data set only two genotypes.

## Authors' contributions

JB analyzed the data set, wrote Fortran programs for calculation of LD, the R code for plotting the figures, and drafted this manuscript, MS developed the program for haplotype reconstruction and FSS designed and supervised this study. All authors reviewed this manuscript.

## Supplementary Material

Additional file 1 **Frequency distribution of minor allele frequency of all SNPs before editing**. Click here for file

Additional file 2 **Frequency distribution of distance between adjacent SNPs located on autosomes**. Click here for file

Additional file 3 **Frequency distribution of distance between adjacent SNPs located on the chromosome X**. Click here for file

Additional file 4 **Decay of linkage disequilibrium (r^2^) with distance of misplaced SNPs (before and after correcting SNP location)**. The titles indicate chromosome number, name and physical location of the misplaced SNP. The even figures represent a decline of LD when location of the misplaced SNP was corrected. Click here for file
