# Supplementary figures and images for: Characteristics of linkage disequilibrium in North American Holsteins
Source: BMC Genomics. 2010 Jul 8;11:421. doi: 10.1186/1471-2164-11-421 (PMC2996949; doi:10.1186/1471-2164-11-421)

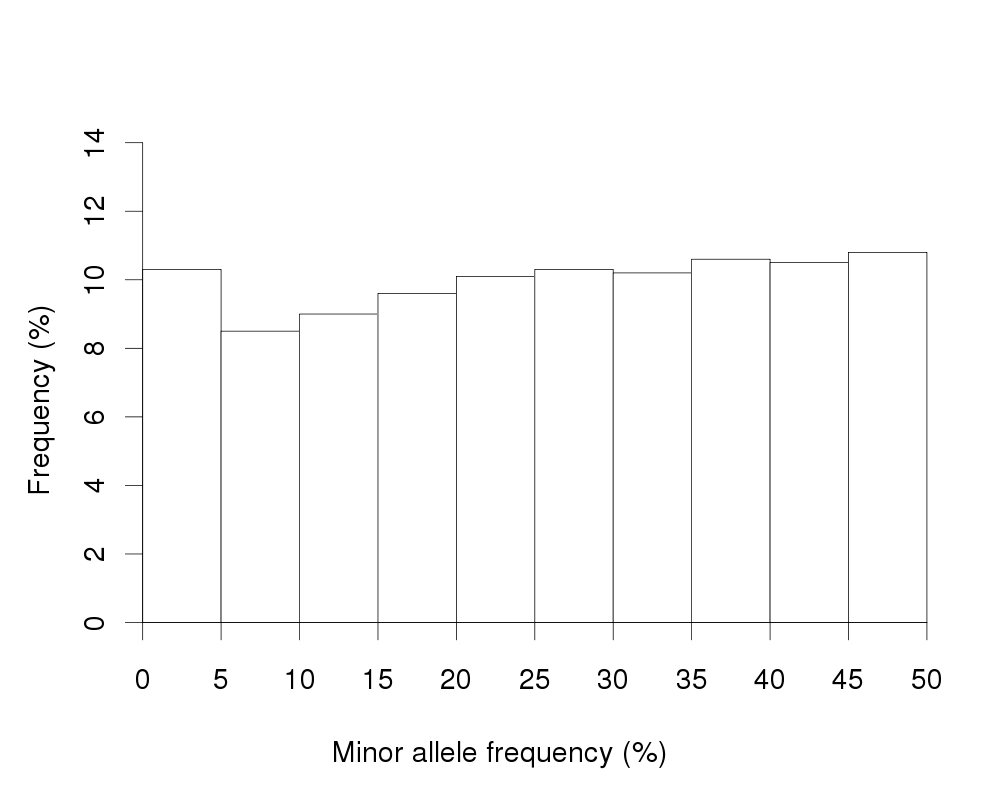

Supplement: Additional file 1 — Frequency distribution of minor allele frequency of all SNPs before editing. [file 1471-2164-11-421-S1.PNG]

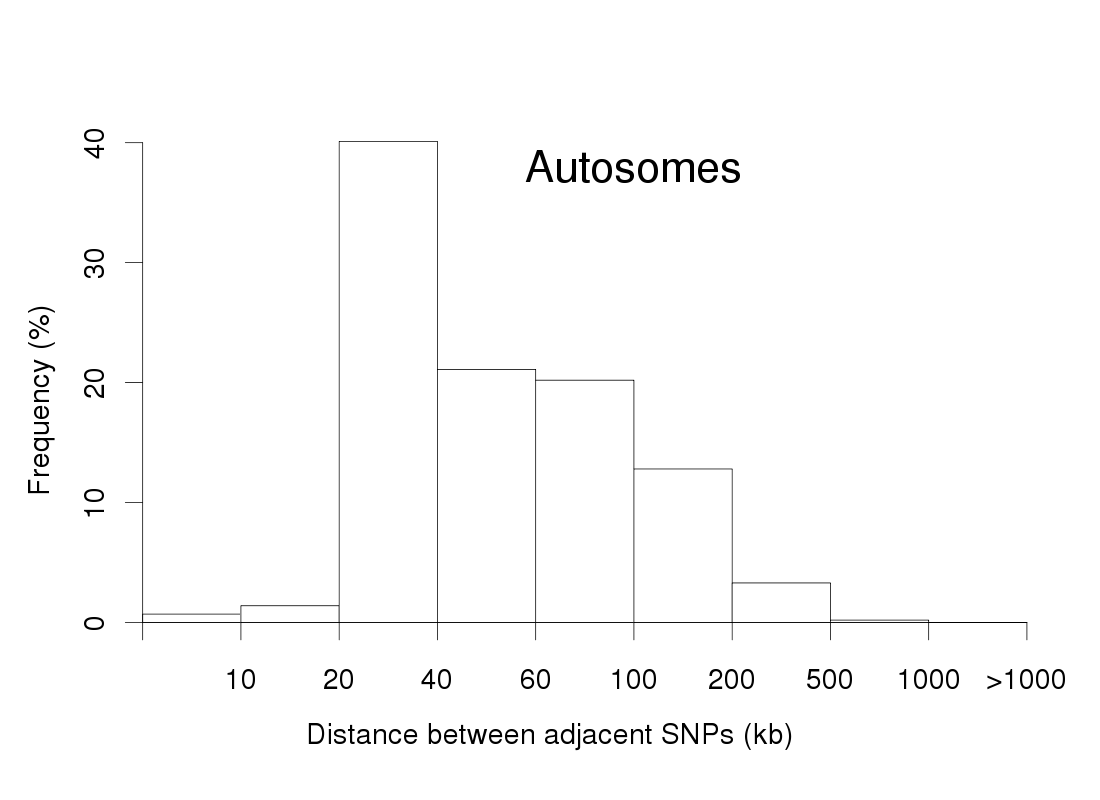

Supplement: Additional file 2 — Frequency distribution of distance between adjacent SNPs located on autosomes. [file 1471-2164-11-421-S2.PNG]

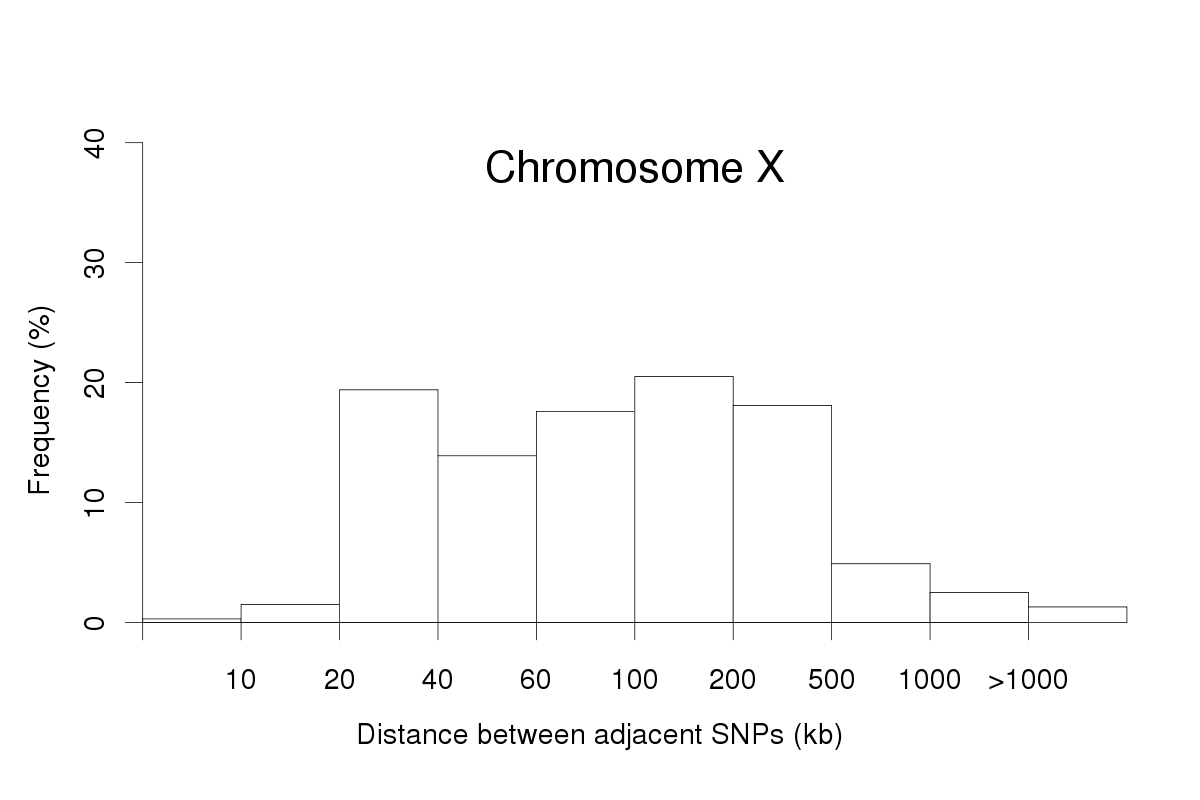

Supplement: Additional file 3 — Frequency distribution of distance between adjacent SNPs located on the chromosome X. [file 1471-2164-11-421-S3.PNG]

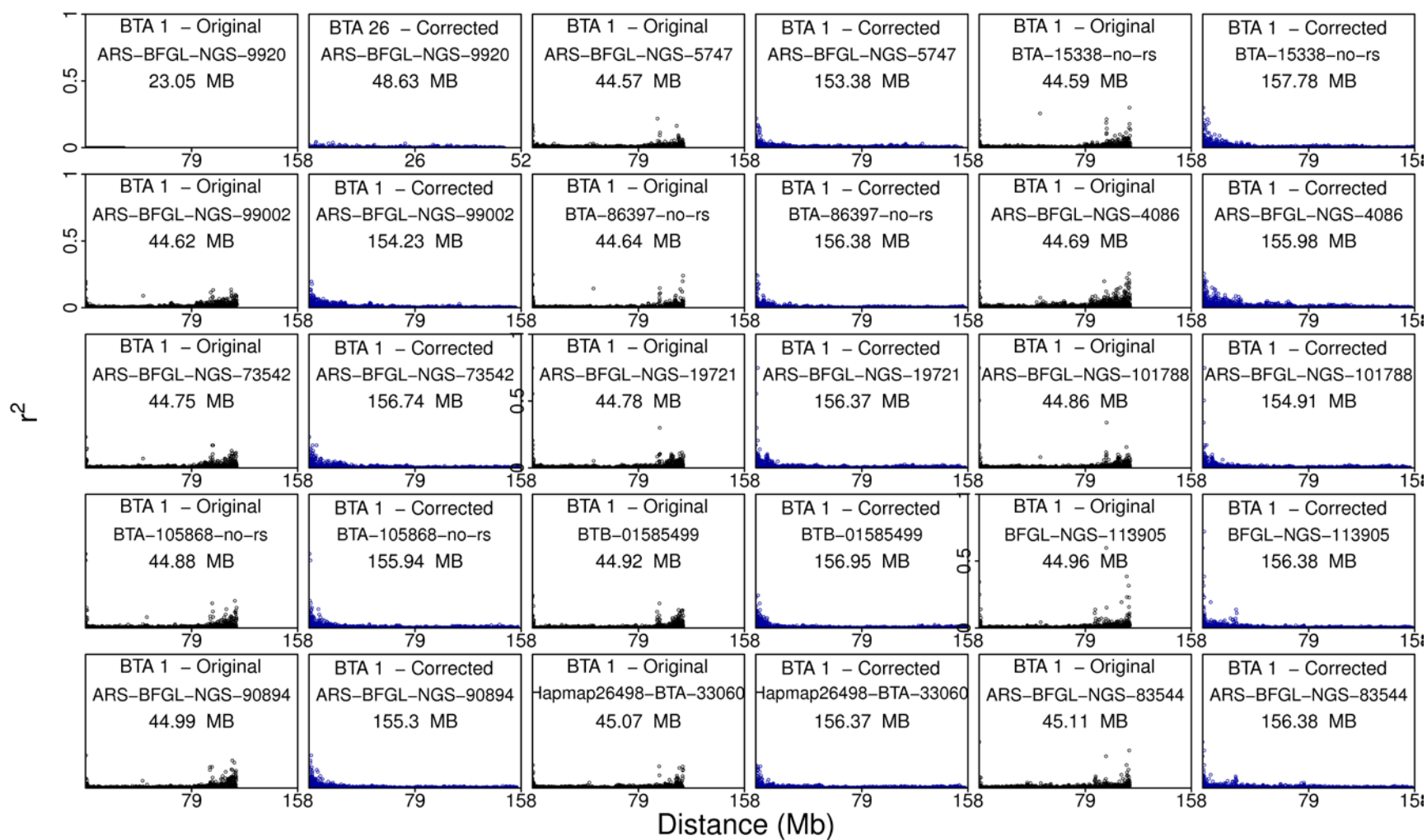

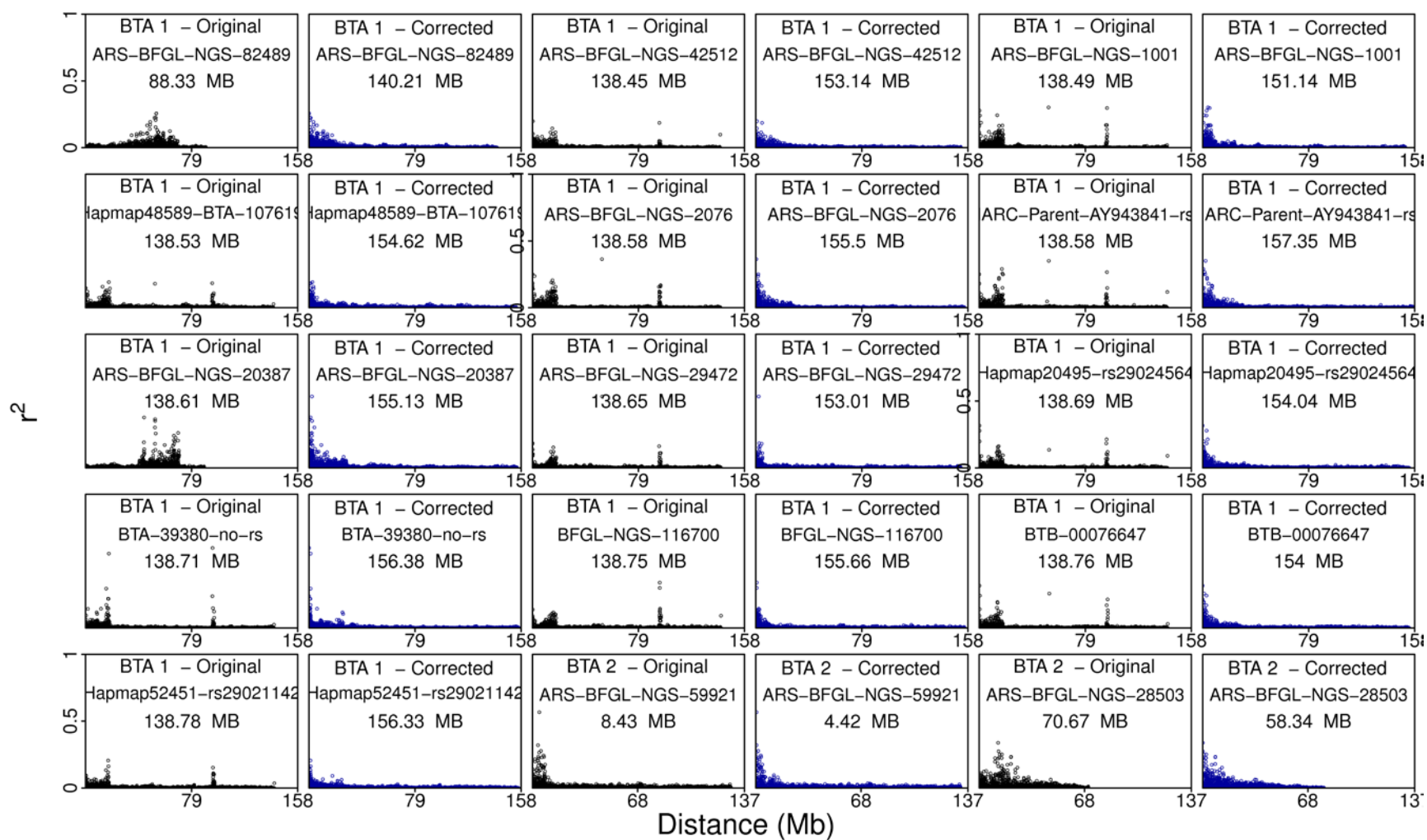

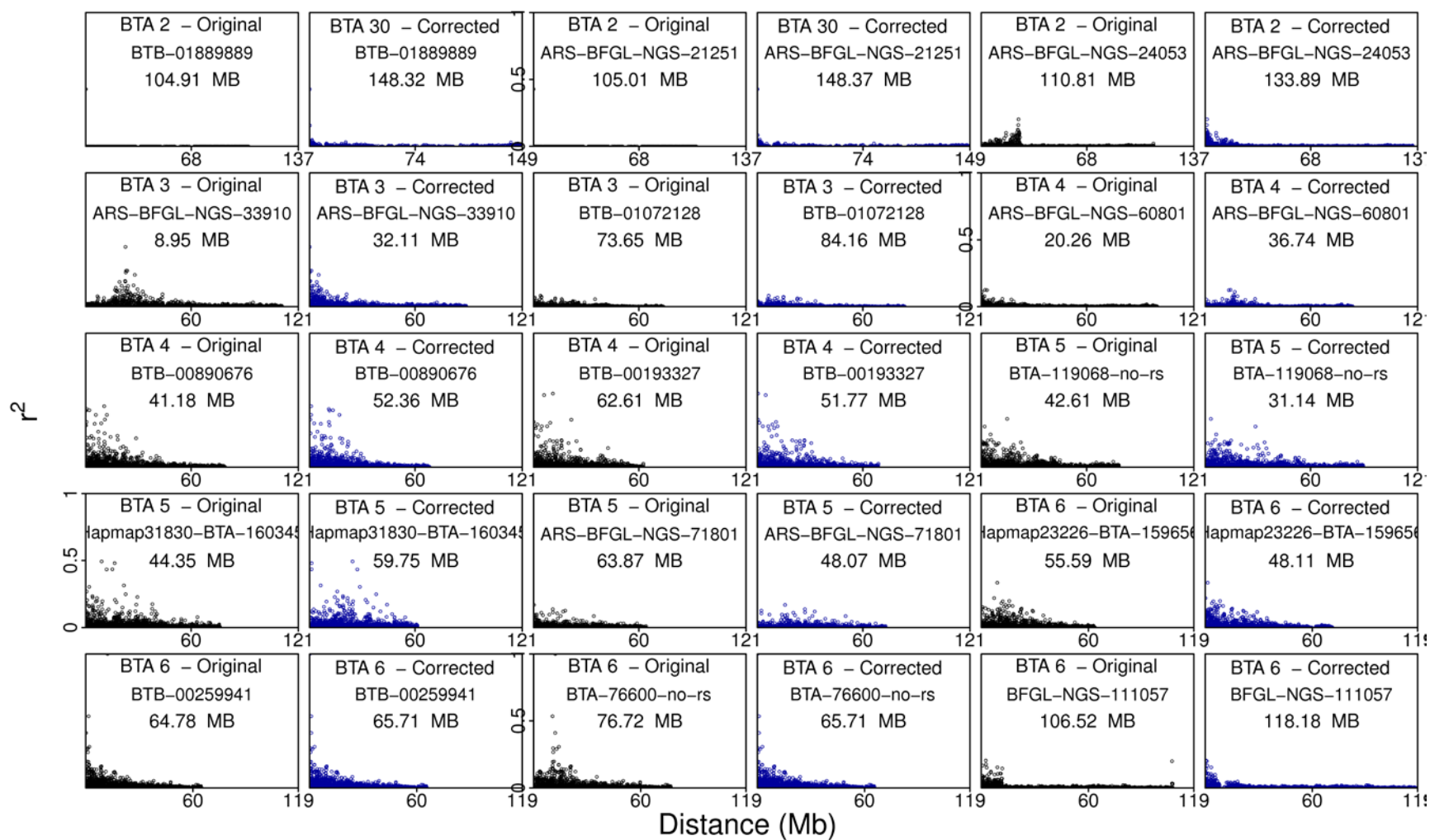

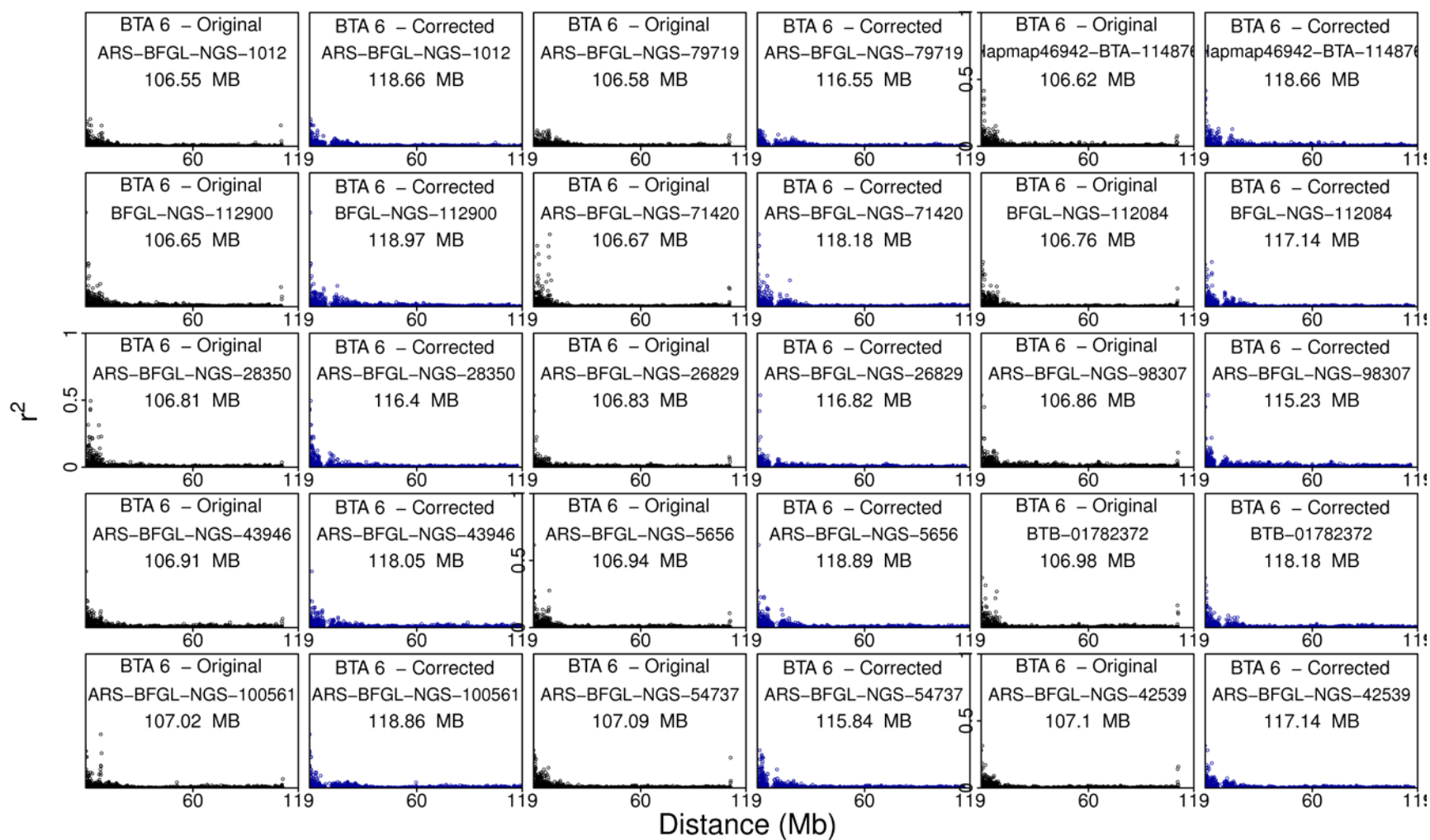

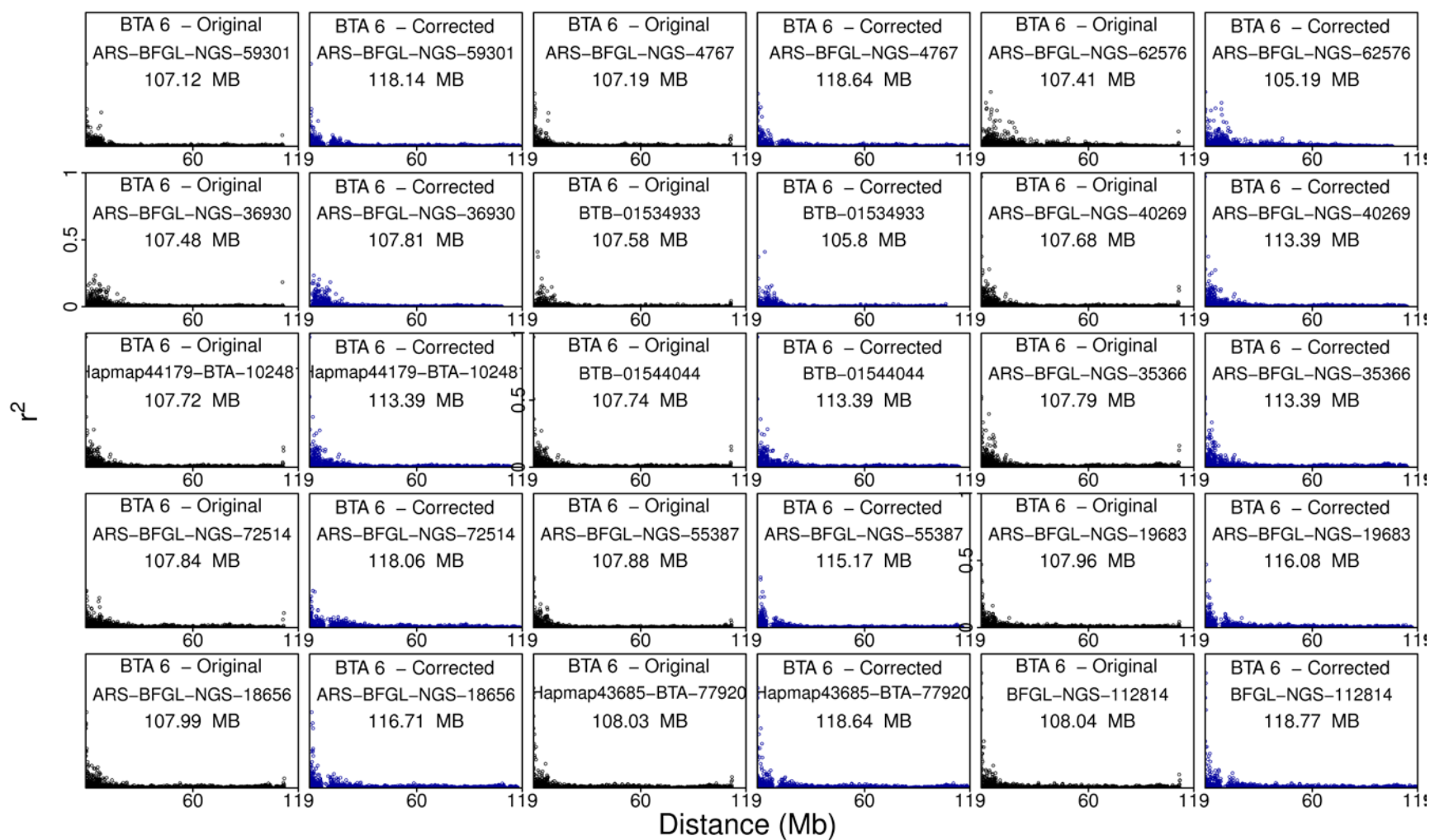

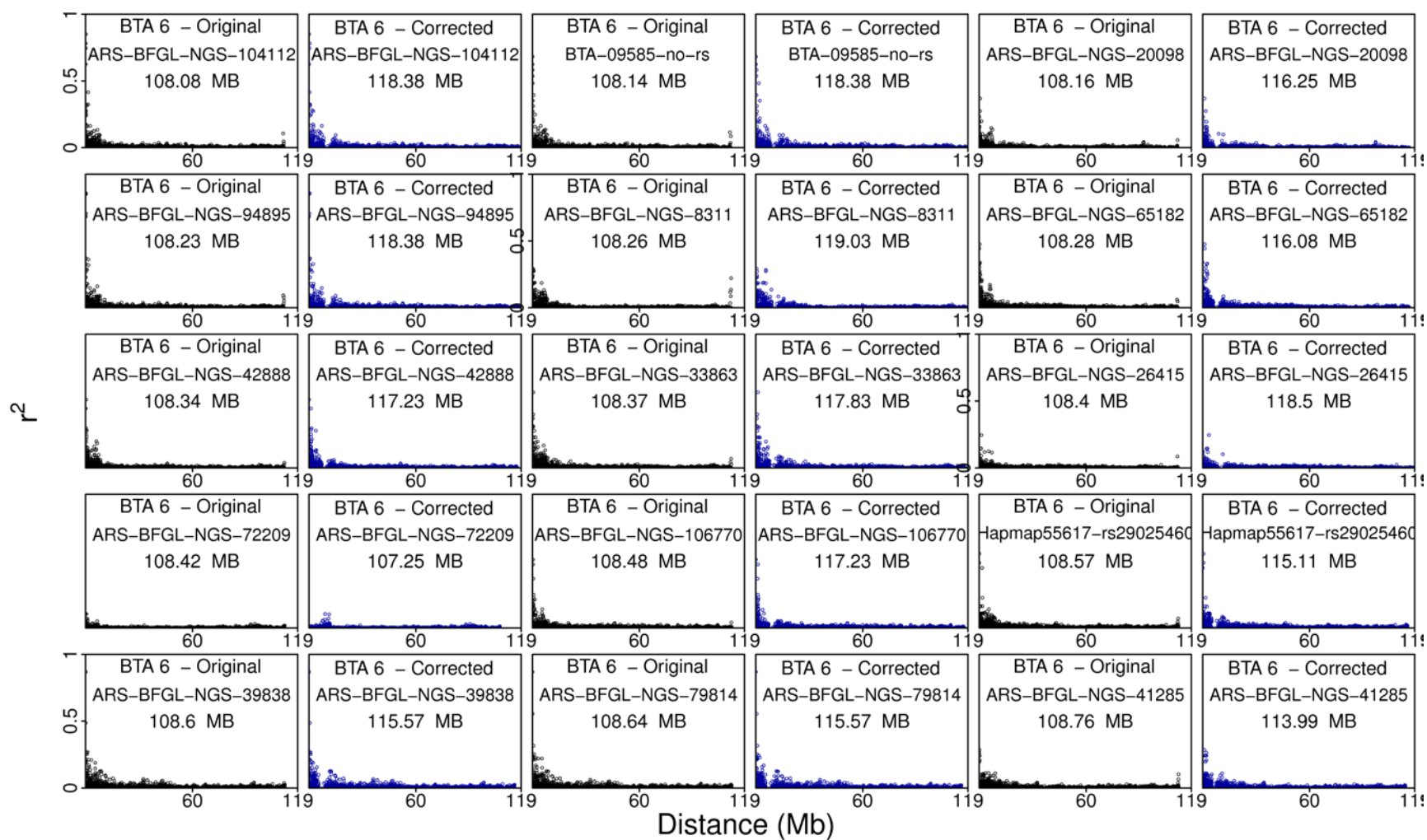

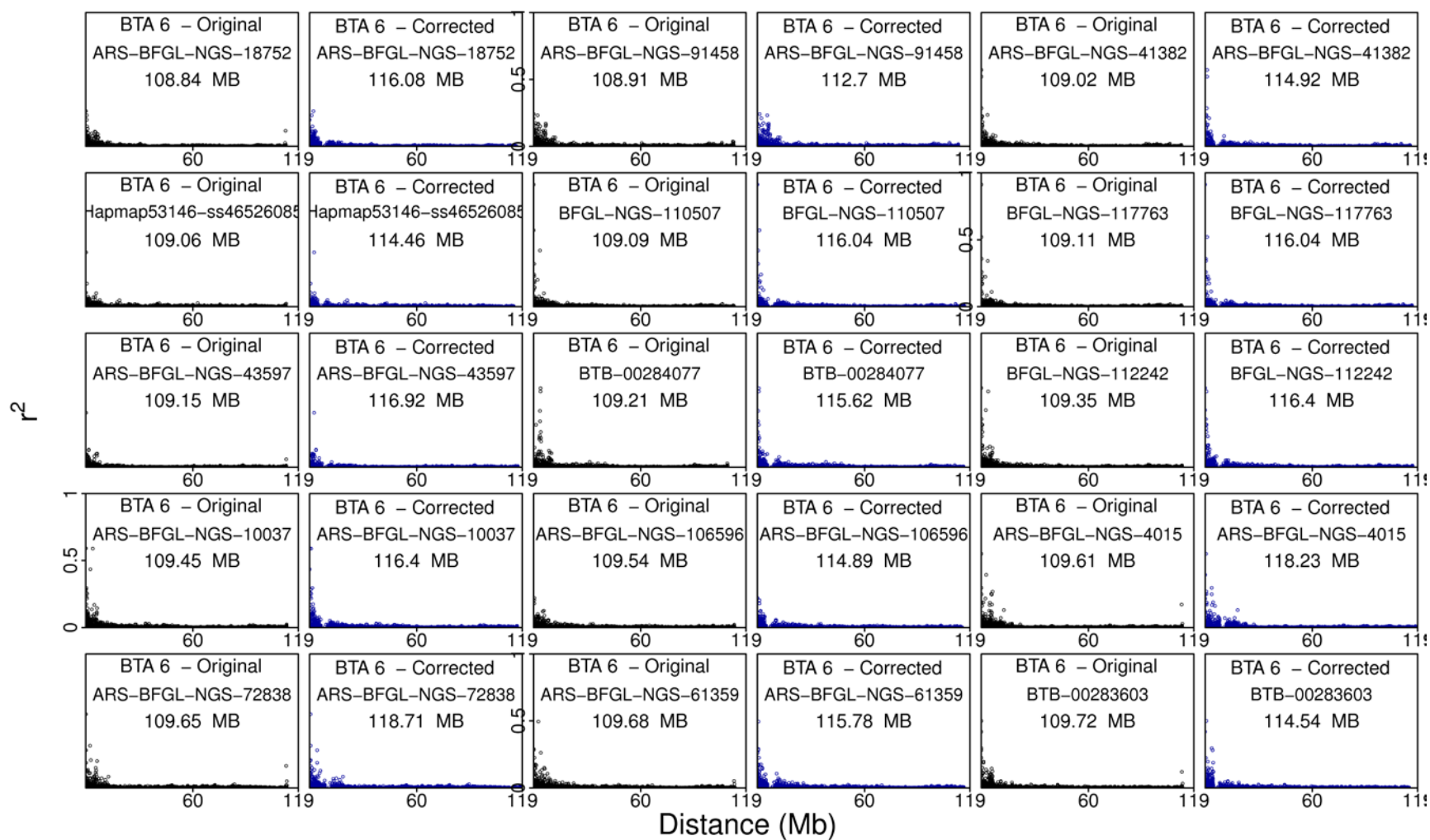

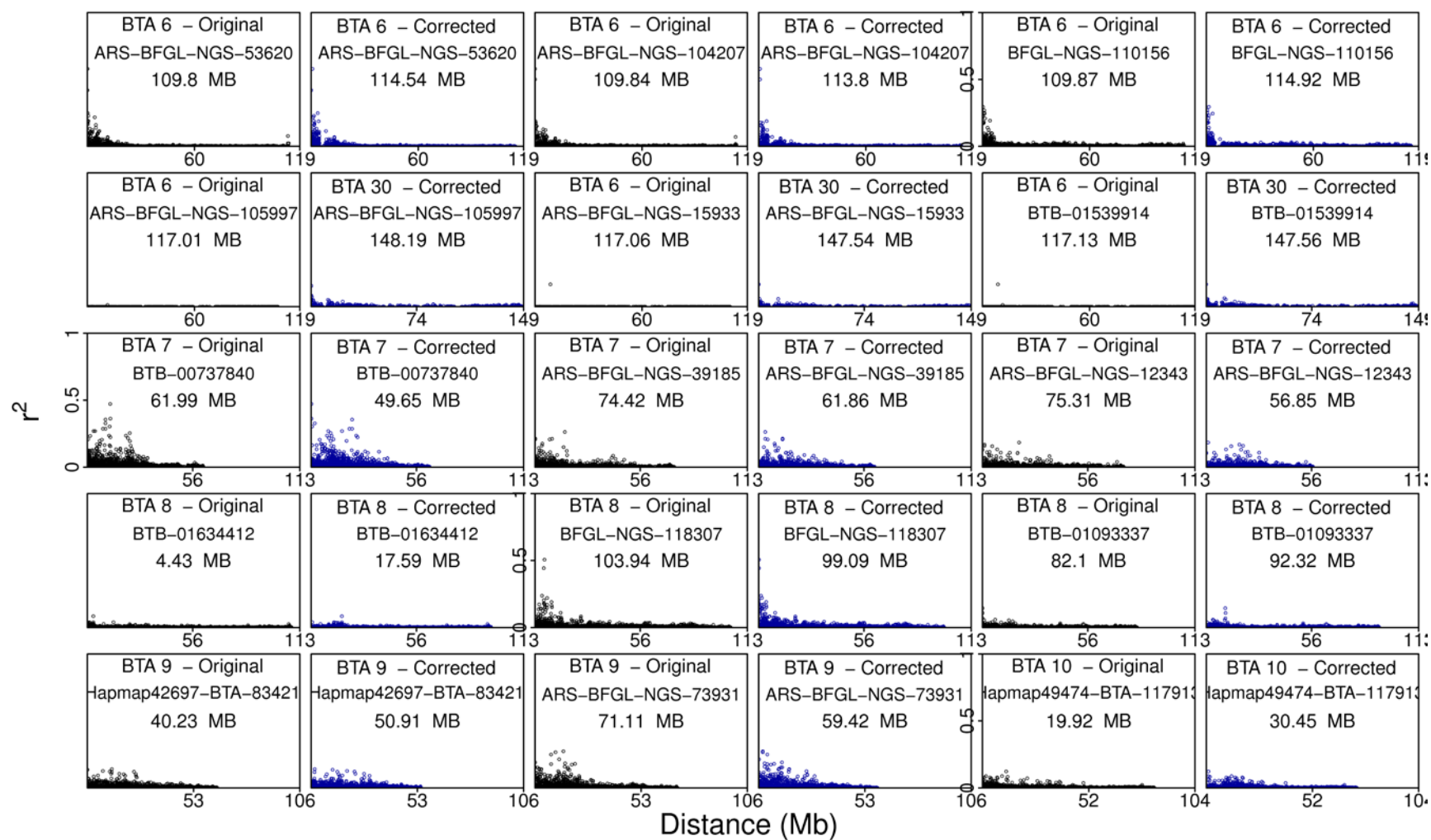

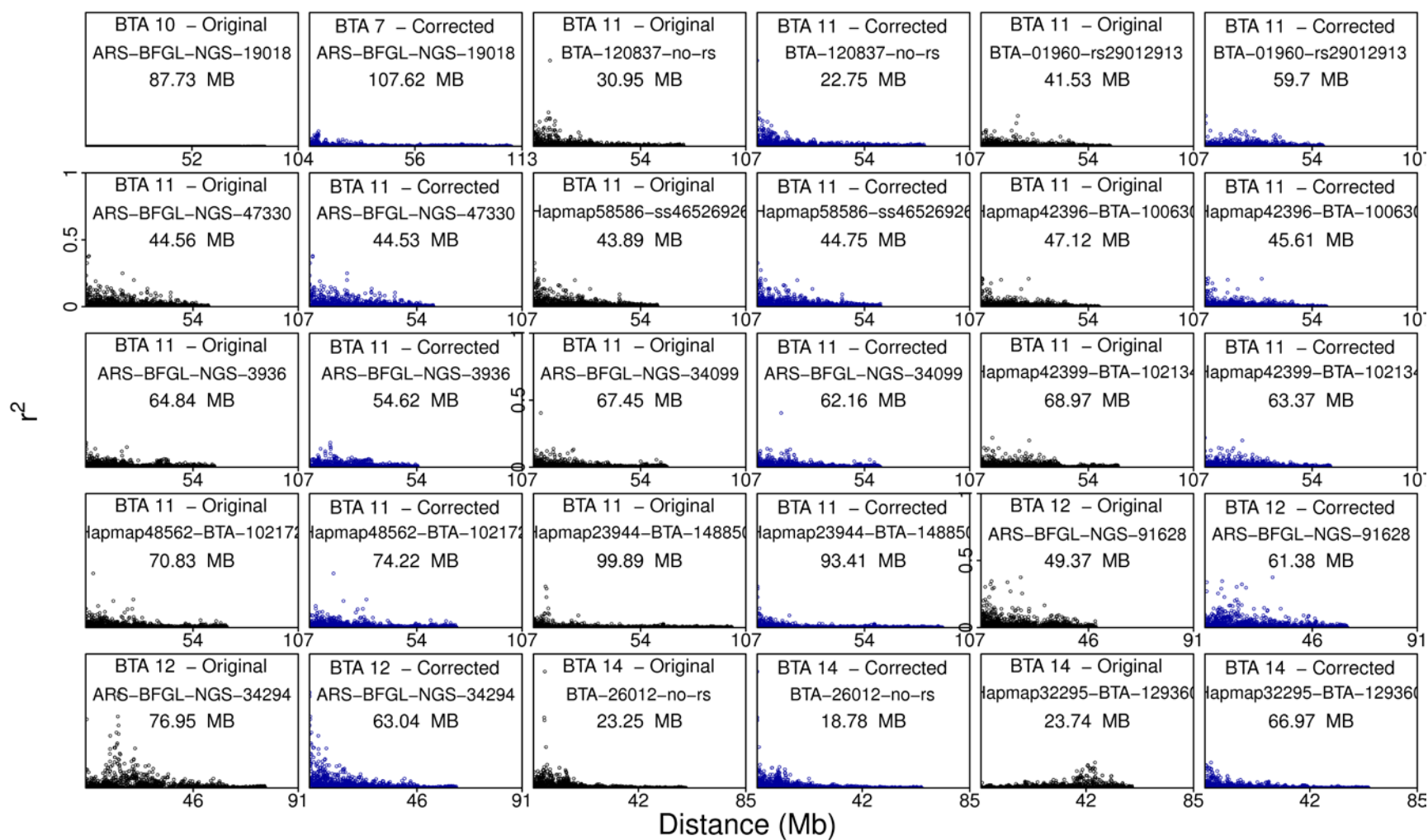

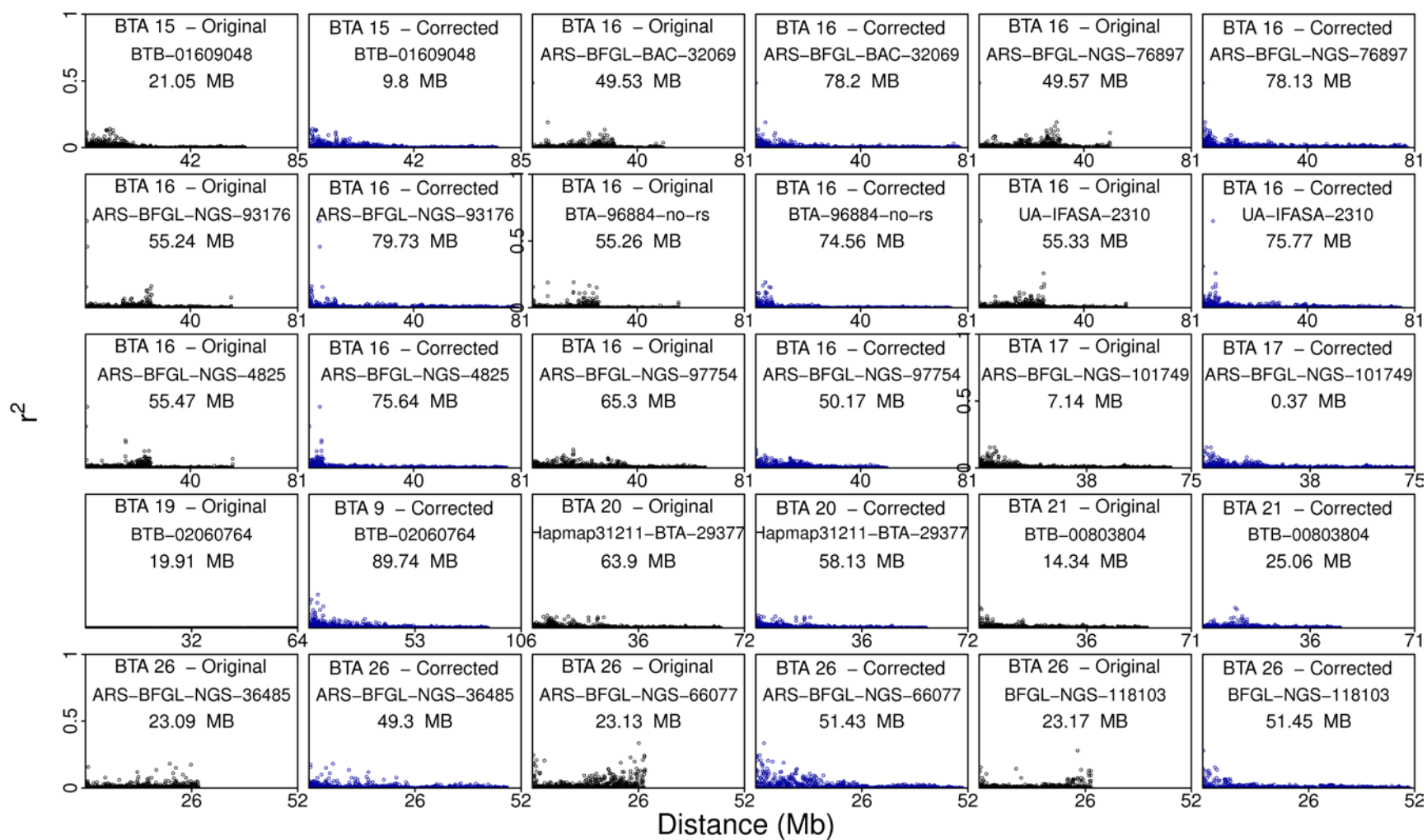

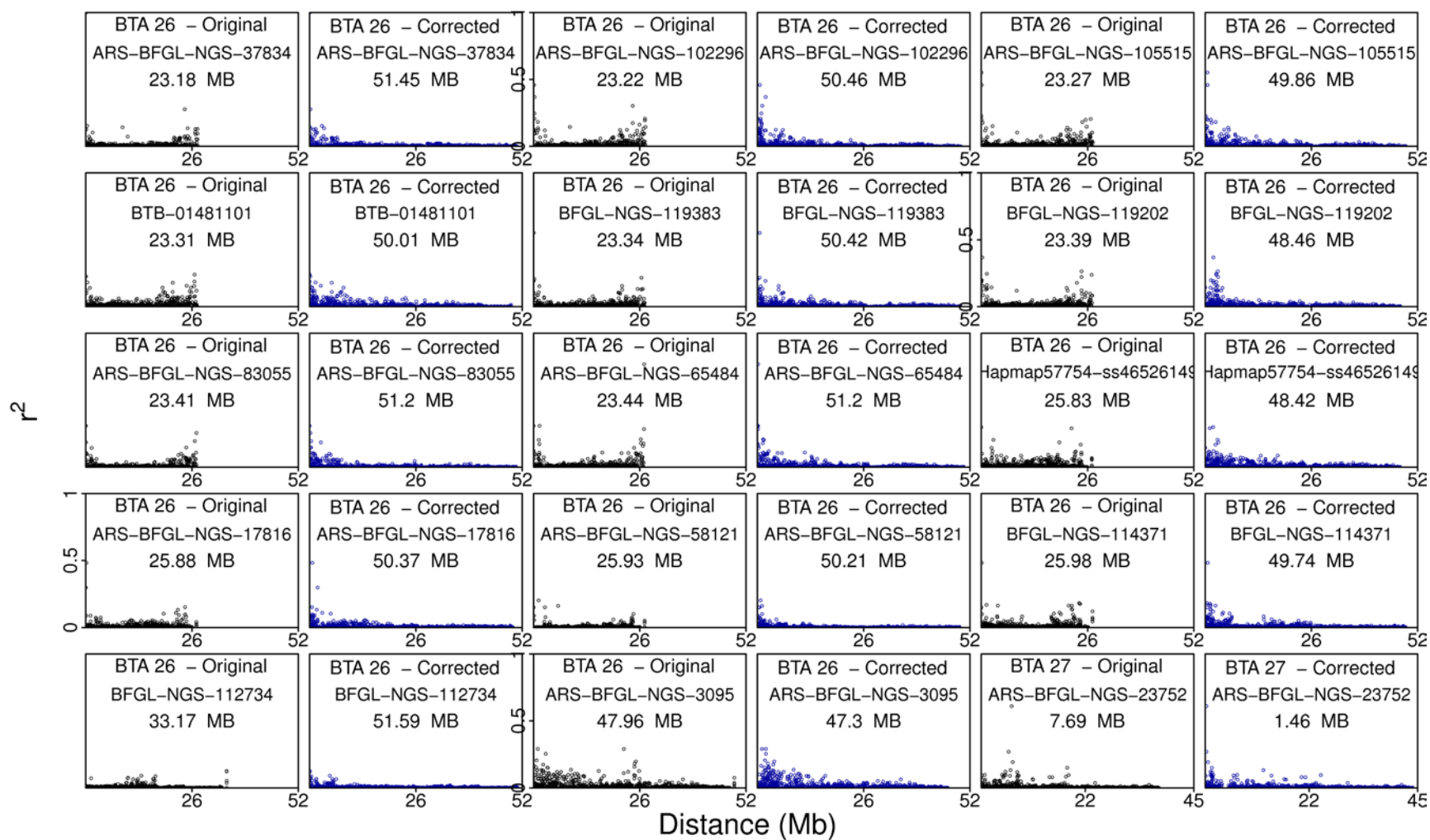

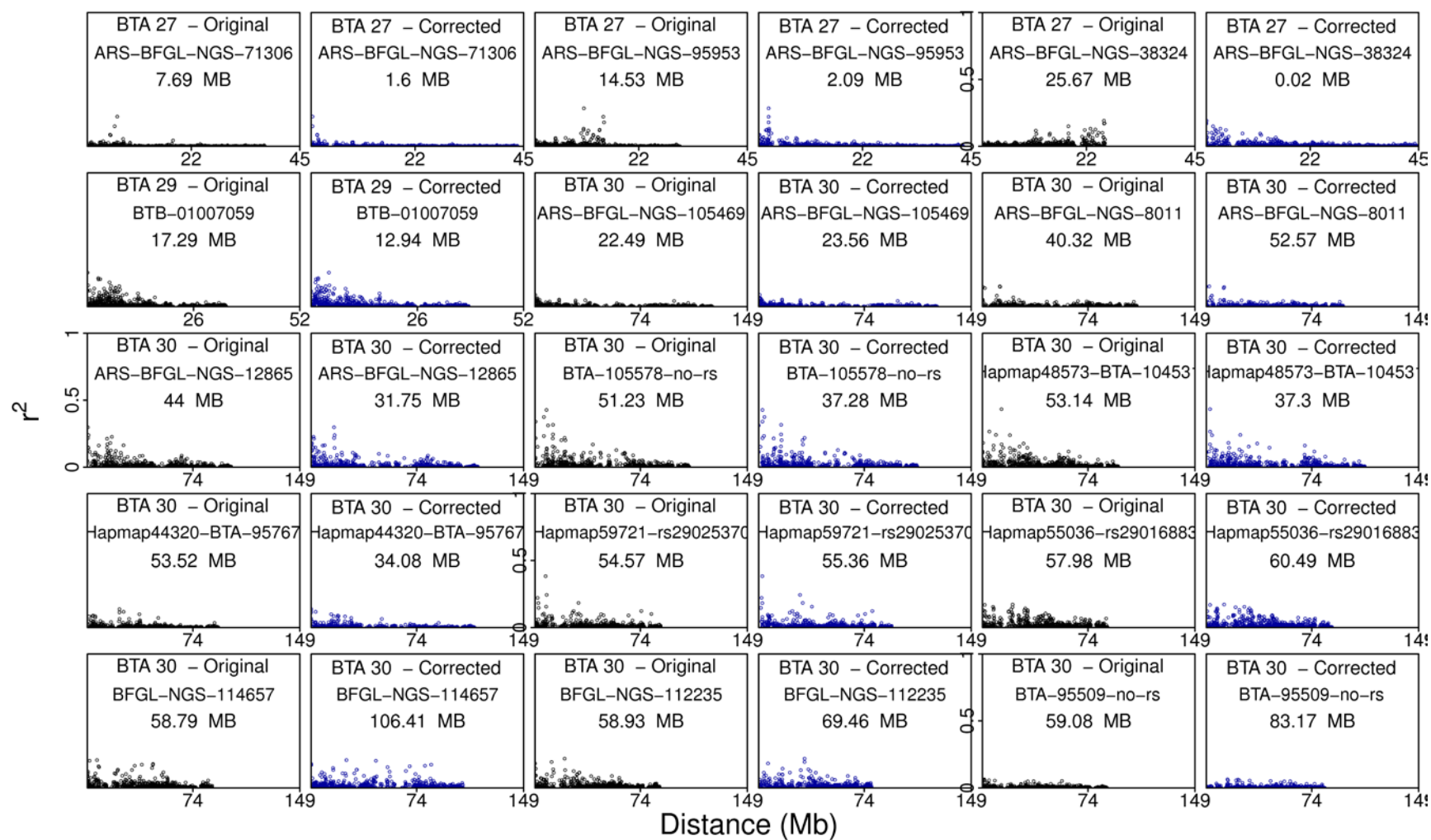

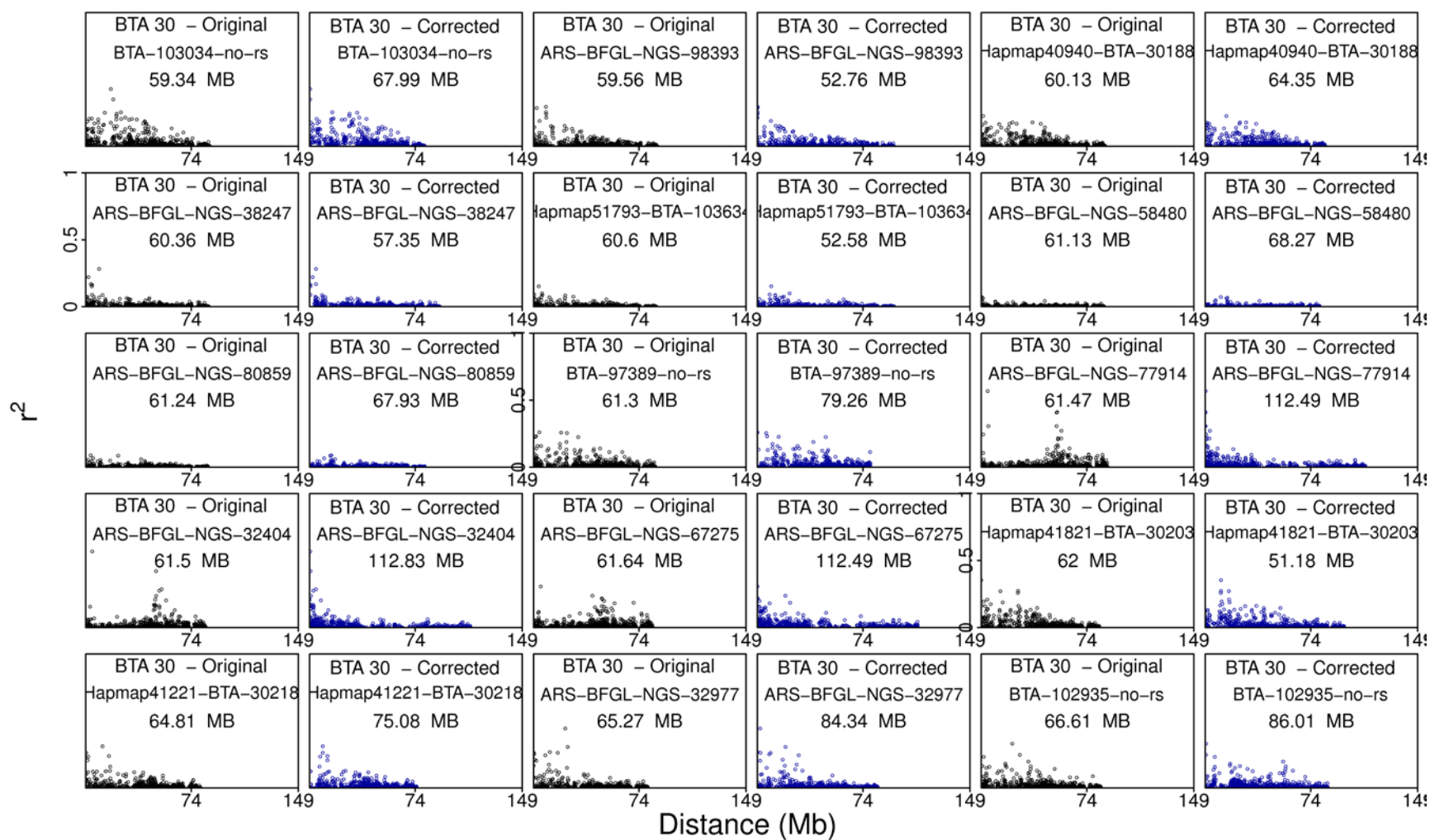

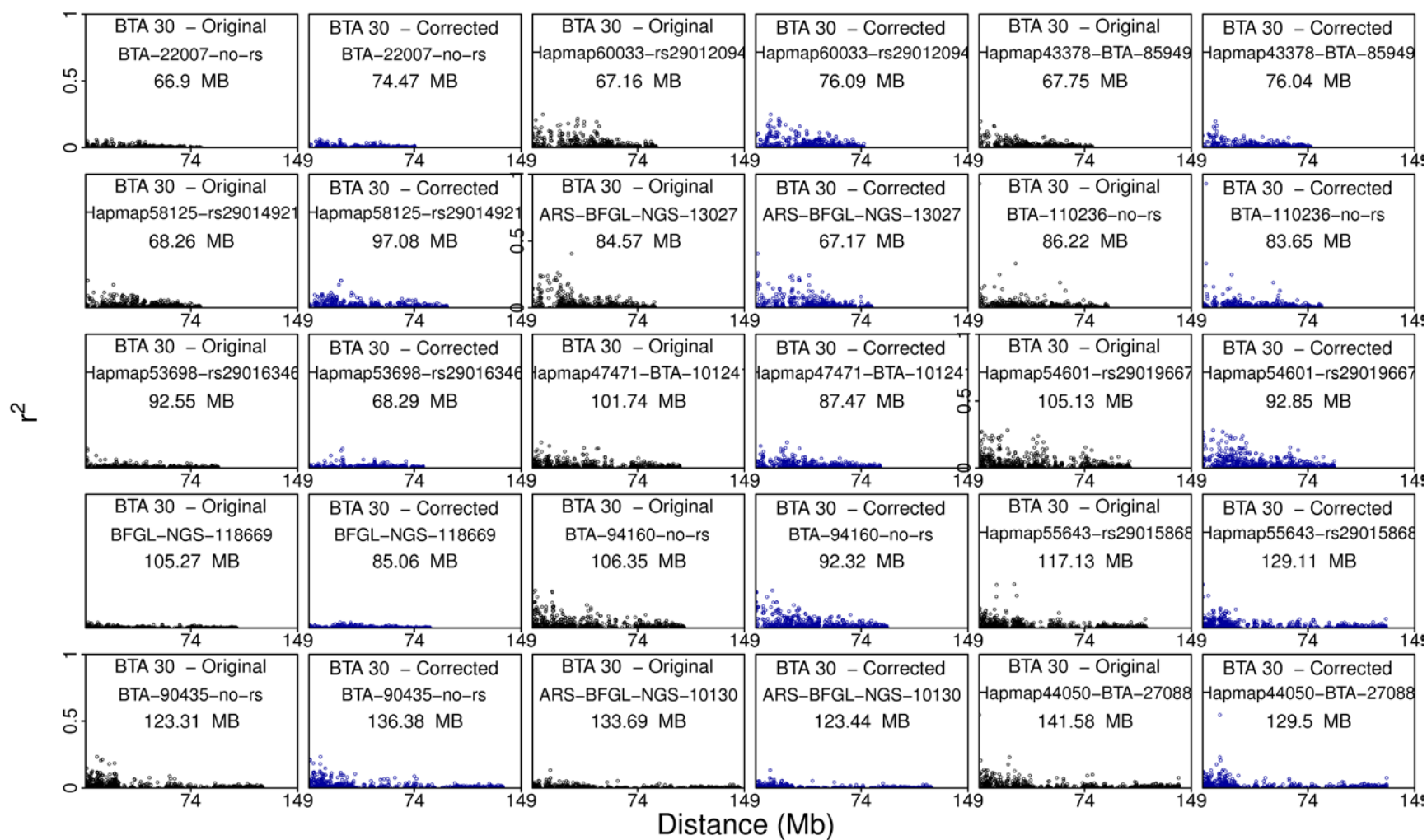

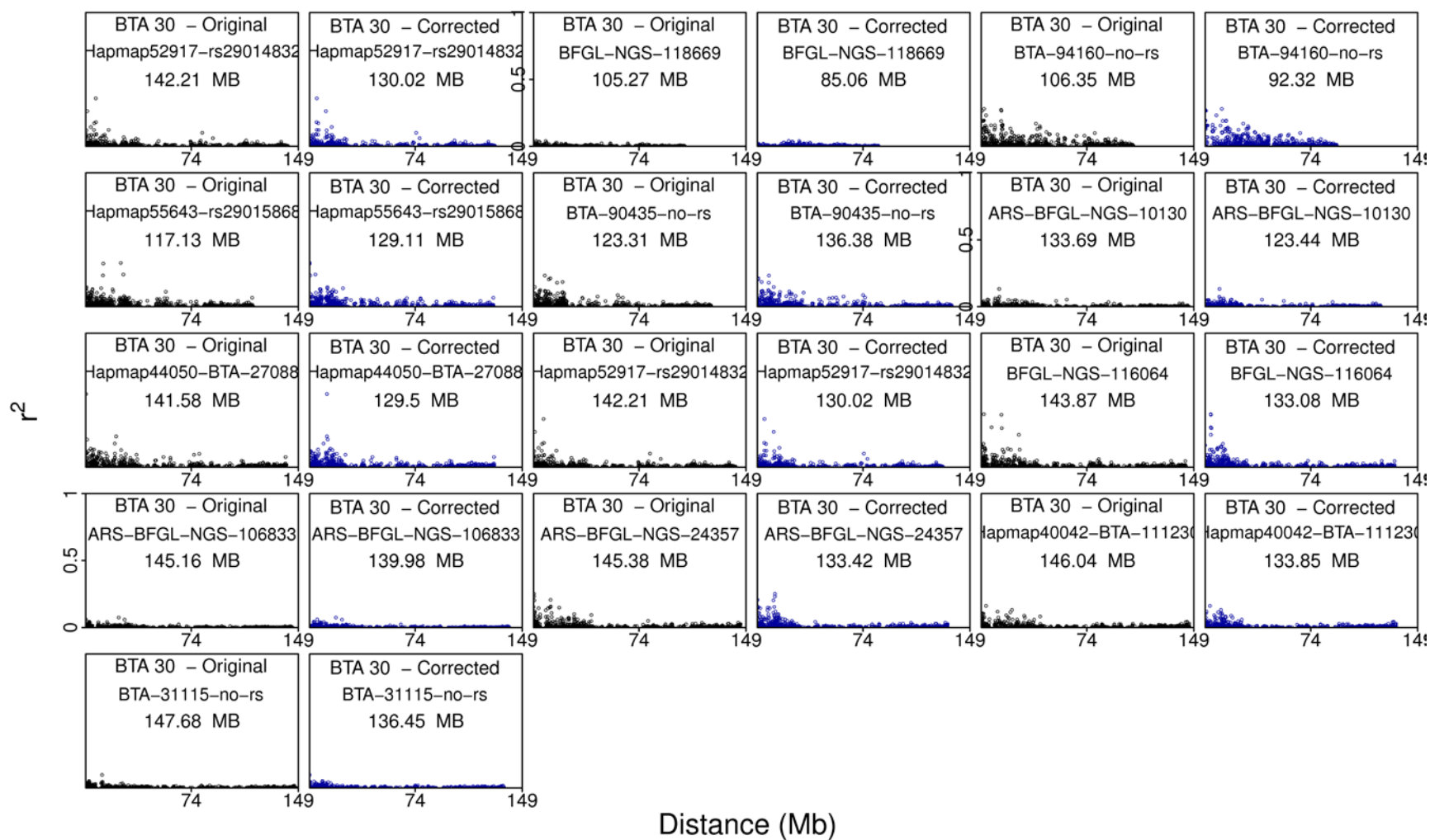

Supplement: Additional file 4 — Decay of linkage disequilibrium (r2) with distance of misplaced SNPs (before and after correcting SNP location). The titles indicate chromosome number, name and physical location of the misplaced SNP. The even figures represent a decline of LD when location of the misplaced SNP was corrected. [file 1471-2164-11-421-S4.PDF]
